# New genetic regulators question relevance of abundant yolk protein production in *C. elegans*

**DOI:** 10.1038/srep16381

**Published:** 2015-11-10

**Authors:** Liesbeth Van Rompay, Charline Borghgraef, Isabel Beets, Jelle Caers, Liesbet Temmerman

**Affiliations:** 1 Functional Genomics and Proteomics, Department of Biology, KU Leuven, Naamsestraat 59 bus 2465, 3000 Leuven, Belgium

## Abstract

Vitellogenesis or maternal yolk formation is considered critical to the reproduction of egg-laying animals. In invertebrates, however, most of its regulatory genes are still unknown. Via a combined mapping and whole-genome sequencing strategy, we performed a forward genetic screen to isolate novel regulators of yolk production in the nematode model system *Caenorhabditis elegans*. In addition to isolating new alleles of *rab-35*, *rab-10* and *M04F3.2*, we identified five mutant alleles corresponding to three novel regulatory genes potently suppressing the expression of a GFP-based yolk reporter. We confirmed that mutations in *vrp-1*, *ceh-60* and *lrp-2* disrupt endogenous yolk protein synthesis at the transcriptional and translational level. In contrast to current beliefs, our discovered set of mutants with strongly reduced yolk proteins did not show serious reproduction defects. This raises questions as to whether yolk proteins *per se* are needed for ultimate reproductive success.

In vertebrates, the hypothalamus-pituitary-gonad (HPG) axis is a regulatory system responsible for the neuroendocrine control of reproduction. One downstream process regulated by this HPG axis in oviparous females is the synthesis of egg-yolk proteins by the liver, which are required for oogenesis^1^,^2^. Yolk proteins are among the most abundant proteins in eggs^3^,^4^. They are primarily produced by the mother in a tissue outside the gonad, are heavily glycosylated and have lipid-binding properties^3^. As such, they provide essential nutrients to the eggs to support embryonic development^4^,^5^,^6^. Most invertebrate species also rely on vitellogenesis, i.e. yolk build-up in the maturing oocytes, for reproduction. To date, only scattered pieces of information are available on the (hormonal) regulation of protostome yolk protein gene expression in response to environmental conditions^4^,^7^,^8^,^9^. It can be expected that genetic control of reproduction in invertebrates will - to a certain extent - be similar to the vertebrate system, as supported by homologous gene sequences found in distinct species. Yet, many invertebrate-, clade- or species-specific factors are thought to exist as well - e.g. depending on distinct reproductive cycles. To explore genetic determinants of invertebrate yolk protein production and their evolutionary conservation, we relied on the nematode model species *Caenorhabditis elegans* and its powerful genetic toolbox, which readily enables the identification of new mutations in key genes and the dissection of molecular genetic pathways.

Putative orthologues of the components of the vertebrate HPG axis have previously been reported for the worm^10^,^11^,^12^,^13^, giving first indications of evolutionary conservation of at least a part of the reproductive control system. Starting from an unbiased screen for mutants defective in *vit-2* (vitellogenin) gene expression as visualized by a *gfp* reporter gene, we here report on three molecular players (i.e. VRP-1, CEH-60 and LRP-2) previously unknown to regulate yolk protein synthesis. We further explored the transcriptional interdependency between these regulators and discuss the unexpected low impact of massive yolk protein production on overall reproductive success.

## Results

### Genetic ethyl methanesulfonate (EMS) screen

The *C. elegans* genome harbours six *vit* genes, all encoding yolk proteins^14^,^15^. These are subdivided into two classes - YP170 and YP88/115 - named according to their approximate molecular weight^15^,^16^,^17^. We created a reporter strain expressing a functional fluorescent VIT-2(YP170B)::GFP fusion protein which is, like endogenous yolk, transported from the intestine into the growing oocytes^6^,^18^,^19^,^20^,^21^.

Starting from this strain, roughly 7,500 EMS-mutagenized haploid genomes were manually analysed during a phenotypic selection step based on aberrant *gfp* expression. This ranged from complete loss of the *gfp* signal (five mutants) over reduced *gfp* expression (one mutant) to abnormal accumulation of GFP (three mutants) (Table 1, Supplementary Fig. S1). The latter three display a typical receptor-mediated endocytosis phenotype, noticeable by abnormal accumulation of fluorescence in the body cavity, outlining the internal organs, with little or no fluorescence in the oocytes and embryos^6^.

### Mapping and allele characterization

To elucidate the phenotype-causing recessive mutations, we relied on a one-step WGS and SNP-based mapping procedure originating from a successful approach in plants^22^,^23^. We retrieved for each mutagenized sequenced genome an unambiguous map position (Supplementary Fig. S1). WGS of the starting strain allocated transgene *lstIs13 [Pvit-2::vit-2::gfp]* to the 5.90–5.92 Mb region on chromosome X. We next looked into all possible causative variants within each mutant’s mapping region (Supplementary Table S1). Some protein coding sequence variants - i.e. premature stops, frameshifts and splice site variants - are of special interest as a result of their allegedly higher impact on protein function.

We isolated three mutant alleles of genes previously described to be involved in yolk (receptor) endocytic trafficking^24^,^25^, consistent with our observations of their characteristic receptor-mediated endocytosis mutant phenotype. These are a novel missense mutation in *rab-35* (RAB family), a novel premature stop in *rab-10* and a premature stop in *M04F3.2* (Table 1). The isolation of these mutants provides proof of concept of our genetic screen.

Despite the premature stop codon (W222amber) arising in *oac-2* for mutant LSC652, its yolk protein mutant phenotype could not be rescued by introducing the wild-type copy of this gene. This mutant also contains an exonic missense mutation in *tam-1* (tandem array expression modifier), a known transgene silencer^26^ (Table 1). Additional YP170B analysis (see below) confirmed that *tam-1(lst538)* causes reduced expression of the *vit-2::gfp* reporter. Similar false positives have been described for reporter-based screens before^27^,^28^,^29^.

In addition, we isolated five alleles comprising three genes that are so far unknown to play any role in yolk protein regulation. Novel causal protein-changing variants of two genes could be pinpointed in the corresponding mutants by rescuing *vit-2::gfp* expression with genomic wild-type copies of these candidates (Supplementary Fig. S1). We uncovered a premature stop allele and a splice site acceptor variant in *Y54G2A.3*, which we named *vrp-1* (vitellogenin-regulating *Caenorhabditis*-specific protein) (Supplementary Fig. S1, Table 1). According to miR-*abela* analysis^30^, the first intron of the *vrp-1* gene contains a potential functional RNA. We therefore also rescued the *vrp-1(lst461)* strain with a cDNA construct showing that its protein-coding sequence underlies the observed phenotype. Absence of similarities in BLAST searches support its *Caenorhabditis*-specific character. The homology-independent search tool FFPred 2.0^31^ predicts VRP-1 to be involved in DNA-dependent regulation of transcription, further supported by its nuclear localization (see below).

Premature stop and splice site variants were similarly discovered for the *ceh-60* gene (*C. elegans* homeobox) in two mutant strains (Supplementary Fig. S1, Table 1).

A novel nonsense mutation was unveiled in *lrp-2* (low-density lipoprotein receptor related) (Supplementary Fig. S1, Table 1). For this strain, however, we could not obtain a genomic rescue, probably due to its relatively large size (~19 kbp) in combination with the strain’s ‘bag of worms’ phenotype. Because no other high impact type of variant resided in its small mapping region, we decided to use the earlier isolated *lrp-2*(gk556942) null mutant in addition to our *lrp-2*(lst464) mutant to ultimately verify the role of *lrp-2* in the regulation of yolk protein production during later experiments^32^. This relates *lrp-2* to its potential effects on yolk protein levels based on the indirect evidence for the identity of the phenotype-causing mutation in the *lrp-2*(lst464) strain.

Since the newly discovered alleles of *vrp-1*, *ceh-60* and *lrp-2* are of special interest, we backcrossed these strains multiple times to the premutagenized background strain, and verified the molecular lesions by PCR and Sanger sequencing to fully support their robust identification. These strains - i.e. *vrp-1(lst461), vrp-1(lst539)*, *ceh-60(lst466)*, *ceh-60(lst491)*, *lrp-2(lst464)* and *lrp-2(*gk556942) - will collectively be referred to as YPR (yolk protein regulating) mutant strains. YPR mutant strains were also outcrossed to wild type in order to eliminate their *vit-2::gfp* transgenic array.

### Endogenous yolk protein and vit mRNA levels are repressed in YPR mutants

Having obtained novel regulators of *vit-2* gene expression, it can now be asked whether the allelic variants emerging from our screen also have a more general impact on yolk protein production (i.e. YP170 and YP88/YP115) in *C. elegans*.

**Figure 1:**
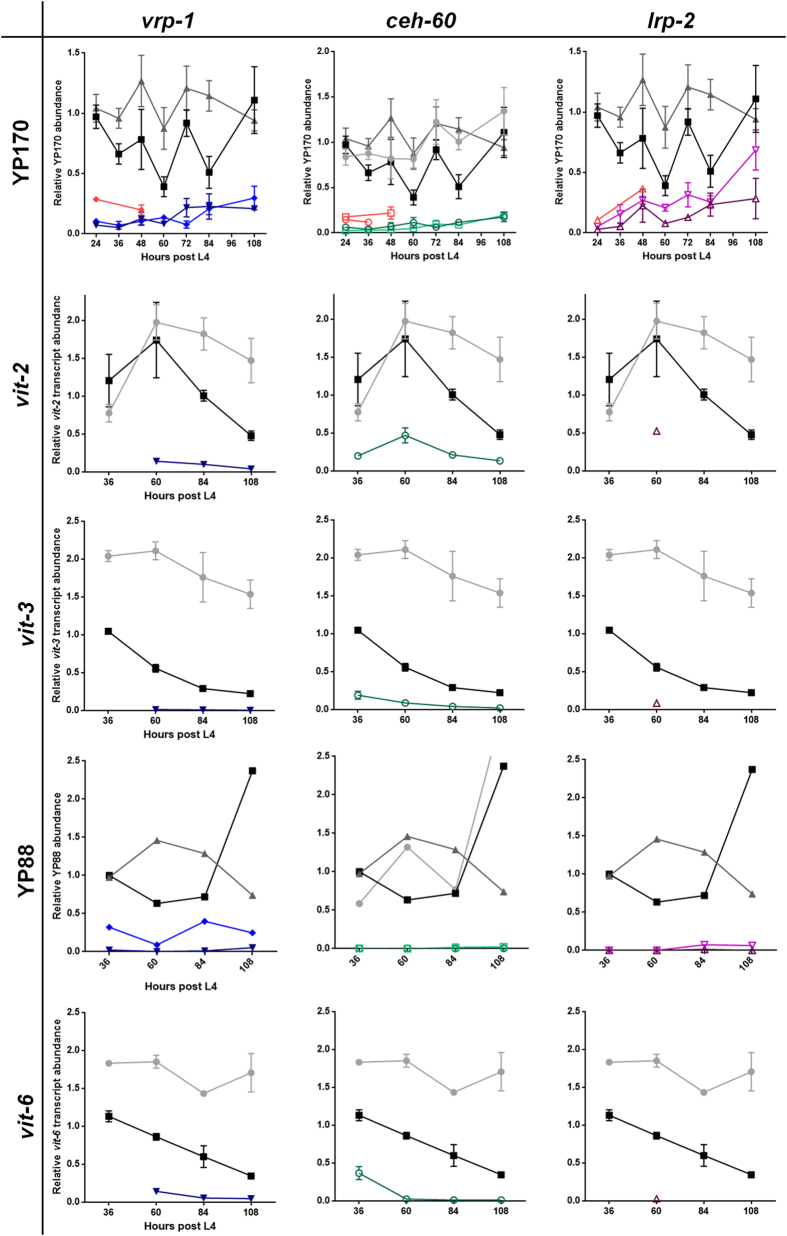
Relative quantification of endogenous yolk protein and *vit* mRNA abundance. YP170 yolk protein levels as analysed by SDS-PAGE were normalized against myosin levels (top row, Supplementary Fig. S2 and S3). Compared to the wild-type (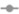), *vit-2::gfp* reporter (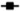) and *tam-1(lst538)* (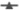) controls, endogenous YP170 levels are considerably lower in all YPR mutant populations, i.e. *vrp-1(lst461)* (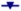), *vrp-1(lst539)* (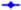), *ceh-60(lst466)* (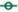)*, ceh-60(lst491)* (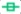), *lrp-2(lst464)* (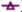) and *lrp-2(gk556942)* (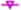). Also in a wild-type background, mutant YPR alleles suppress endogenous YP170 (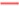). The *vit-2* (second row) and *vit-3* (third row) mRNA expression profiles are consistent with these YP170 yolk protein data (first row). YP88 immunoblot data are expressed relative to each sample’s total protein signal (fourth row; Supplementary Table S2). In sharp contrast to both *ceh-60*, the *vrp-1(lst461)* and both *lrp-2* mutants, the *vrp-1(lst539)* mutant still displays some YP88 immunoreactivity. The underlying *vit-6* (bottom row) mRNA expression profiles again correlate well with these YP88 yolk protein data (Supplementary Table S2). For each indicated time point throughout reproductive development, the mean value of a maximum of three (mRNA) or four (protein) biologically independent measurements ± SEM is plotted and connected to assist in overall profile evaluation (see also Supplementary Table S2).

Compared to the control strains, endogenous YP170 yolk protein levels were strongly reduced or nearly absent in all YPR mutants throughout reproductive adulthood (Fig. 1, Supplementary Fig. S2 and S3, Supplementary Table S2). Whereas this effect was even more pronounced at the YP170B::GFP fusion protein levels (Supplementary Fig. S2 and S3), it holds for endogenous yolk levels in a wild-type background - i.e. independent of the *vit-2::gfp* transgene (Fig. 1, Supplementary Fig. S2, Supplementary Table S2).

Endogenous YP170 levels were not reduced in *tam-1(lst538)*, in contrast to the YP170B::GFP fusion protein levels, as can be expected for a transgene silencer mutation (Fig. 1 and S2A-C, Supplementary Table S2). We selected this mutant as an additional negative control during further analyses.

Endogenous YP170 levels in wild-type and *vit-2::gfp* reporter controls seem to remain stable throughout reproductive adulthood, with higher relative amounts present in wild type. However, YP170::GFP adds to the total YP170 levels of the reporter strain, which overall reaches levels similar to the wild-type endogenous YP170 pool. In addition, YP170::GFP increases throughout reproductive adulthood, an observation that has been reported by others for endogenous YP170^33^.

We further monitored endogenous levels of the individual YP88 yolk protein by means of an anti-vitellogenin antibody (Fig. 1 and S2D, Supplementary Table S2)^3^. Throughout reproductive adulthood, the YP88 pool was abundantly detected in the control strains, but severely reduced in *vrp-1(lst539)* and virtually absent in all other YPR mutants. Though post-translational influences cannot entirely be excluded, these results presumably also apply to YP115, since it originates from the same precursor polypeptide. YP115 cross-reactivity of the YP88 antibody supports this statement (Supplementary Fig. S2).

To verify whether the differences observed at the protein level indeed resulted from decreased *vit* gene expression, we measured relative expression levels of *vit*-*2*, *-3* (~YP170) and *-6* (=YP88/YP115) in representative YPR mutants (i.e. *ceh-60(lst466)*, *vrp-1(lst466)* and *lrp-2(lst464)*), in comparison with the control strains. The other *vit* genes contributing to the YP170 pool - i.e. *vit-1*, *-4* and *-5* - were not analysed since we (Supplementary Fig. S2) and others have observed *vit-1* to *-5* to be expressed at similar levels^33^,^34^.

Throughout fertile adulthood, *vit-2*, *-3* and *-6* transcript levels are overall reduced in all studied YPR mutants as compared to controls (Fig. 1, Supplementary Table S2). Yet, this effect is less pronounced when comparing with the reporter strain only. This can be attributed to the much faster decrease in *vit-2*, *-3* and *-6* transcript levels towards the end of reproductive adulthood in the reporter control strain compared to wild type. Such a decline in *vit-2* transcript levels has also been reported by others in a wild-type background^33^.

**Figure 2:**
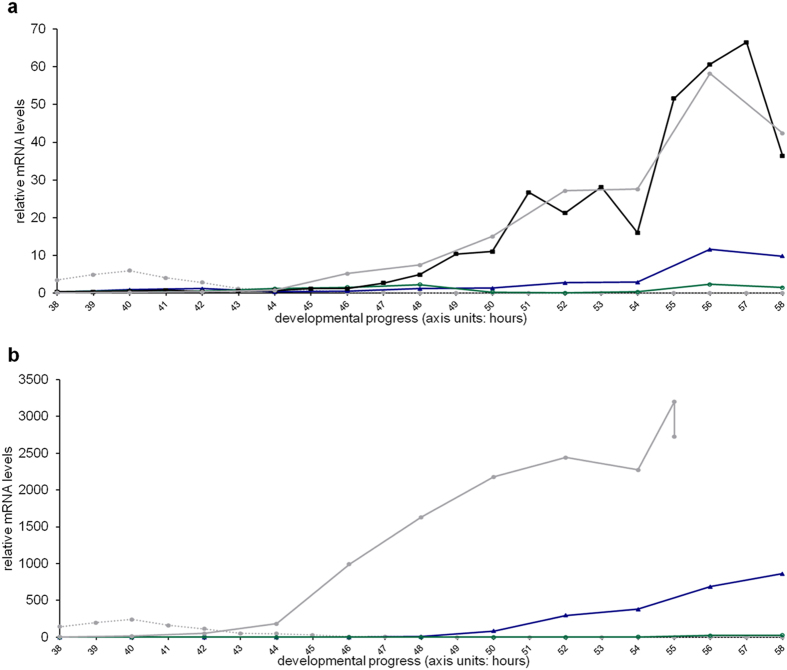
*vit-2* and *vit-6* gene expression are not properly switched on in YPR mutants. Light grey dotted line: wild-type *lin-42* profile to assist in developmental timing evaluation^111^, wild type (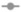), *vit-2::gfp* reporter control (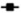), *vrp-1(lst461)* (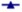), *ceh-60(lst466)* (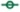), all quantified as of the beginning of the L4 stage (profiles connect single measurements). (**a**) *vit-2* mRNA levels of wild-type and *vit-2::gfp* reporter animals are heavily up-regulated upon becoming young adults, whereas this is not convincingly so in *vrp-1(lst461)* and nearly not at all in *ceh-60(lst466)* mutants. The *vit-2* mRNA pool in all except wild-type animals also contains mRNA derived from the translational *vit-2::gfp* reporter construct. (**b**) In wild type, *vit-6* up-regulation initiates slightly before that of *vit-2*, and covers an impressively larger dynamic range. *vit-6* is the only YP88/YP115-providing gene, whereas the other five *C. elegans vit* genes can contribute to the YP170 pool. Also here, a very moderate (*vrp-1(lst461)*) - to no (*ceh-60(lst466)*) *vit-6* up-regulation is observed in the selected YPR mutants. The *lin-42* profile in panel b has been multiplied by 40 as compared to all other figures in this manuscript to facilitate visibility.

In addition, we verified the proper initiation of *vit-2* expression in the reporter strain, which happens with the same timing and to the same extent as wild-type (endogenous only) *vit-2* expression initiation (Fig. 2). In contrast, the *vrp-1* and *ceh-60* mutants fail to substantially up-regulate their *vit-2* and *vit-6* expression, even though it is initiated at the same time - though moderately (*vrp-1(lst461)*) or nearly not (*ceh-60(lst466)*).

Taken together, these data show that on top of *vit-2::gfp* reporter gene suppression (Supplementary Fig. S2), the here isolated YPR mutants display severely reduced endogenous yolk protein and corresponding *vit* mRNA levels.

### Intestinal VRP-1, amphid CEH-60

**Figure 3:**
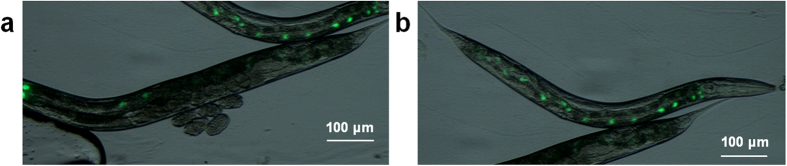
*vrp-1* is expressed in intestinal nuclei. Overlay of the bright field and fluorescence micrograph of (**a**) an egg-laying adult hermaphrodite and (**b**) a larva expressing *vrp-1::gfp*.

To better understand how VRP-1 regulates yolk protein synthesis, we sought to identify its expression pattern. Using a translational *vrp-1::gfp* reporter construct, we observed expression in the intestinal nuclei of both adult and larval hermaphrodites (Fig. 3). Gene expression patterns and stages of *ceh-60*, *lrp-2* and *vit-6* have been determined previously (Supplementary Table S3). Due to its intriguing site of action, we reconfirmed the *ceh-60* expression pattern by confocal microscopy and DiI staining (Supplementary Fig. S4). As reported previously^35^, we could only observe robust *ceh-60* expression in a single pair of amphids, and occasionally weakly so in a second pair.

### Transcriptional regulation in vit-regulatory mutants

**Figure 4:**
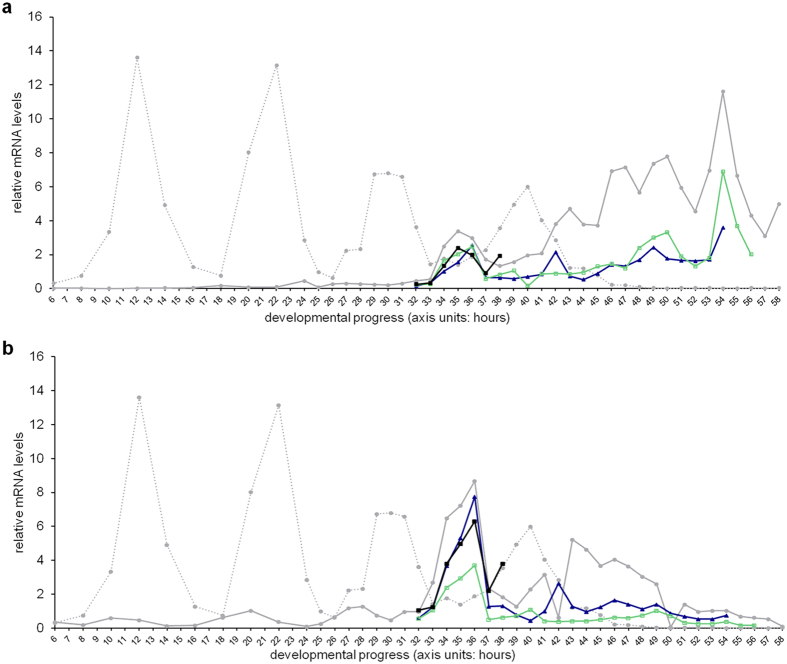
YPR gene profiles reveal critical times during development and possible genetic pathway interdependency. Light grey dotted line: wild-type *lin-42* profile to assist in developmental timing evaluation^111^. (**a**) is *vrp-1*; (**b**) is *ceh-60* expression in the following strains: wild type (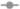), *vit-2::gfp* (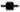), *vrp-1(lst461)* (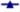) and *ceh-60(lst491)* (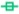) (all profiles connect single measurements). In line with their effects on yolk levels in *C. elegans*, YPR genes are up-regulated in the later part of the developmental cycle (also Supplementary Fig. S5). *vrp-1* levels are affected in *ceh-60* mutants and *vice-versa*.

To examine relevant periods of action during development, as well as to establish possible genetic interdependence in their control of yolk protein expression, relative expression levels of *vrp-1*, *ceh-60* and *lrp-2* genes were measured in *vrp-1(lst461)*, *ceh-60(lst491)*, wild-type and reporter controls. *vrp-1* (Fig. 4a), *ceh-60* (Fig. 4b) and *lrp-2* (Supplementary Fig. S5) transcript levels are initially up-regulated during the L3-L4 wild-type moult, a transcriptional event that seems to be largely unaffected in all mutants under study. During the L4-adult moult, transcription of *vrp-1* and *ceh-60*, but not *lrp-2*, is activated once more in wild-type animals. *ceh-60* transcript levels are down-regulated in *vrp-1(lst461)*, while the reverse is equally true. While *vrp-1* and *ceh-60* transcripts are repressed in their respective mutants, this is not true for *lrp-2* (Supplementary Fig. S5).

### Reproduction seems unaffected by yolk depletion

Generally, *vit* genes are believed to act in a dose-dependent manner^36^. *C. elegans* may thus require all six active *vit* genes for its few intestinal cells to provide sufficient yolk proteins for the production of a large amount of eggs during its short life span^18^,^37^. However, apart from LRP-2 (whose mutants display the ‘bag of worms’ phenotype), malfunctioning of neither VRP-1 nor CEH-60 causes rigorous reproduction defects at first sight.

**Figure 5:**
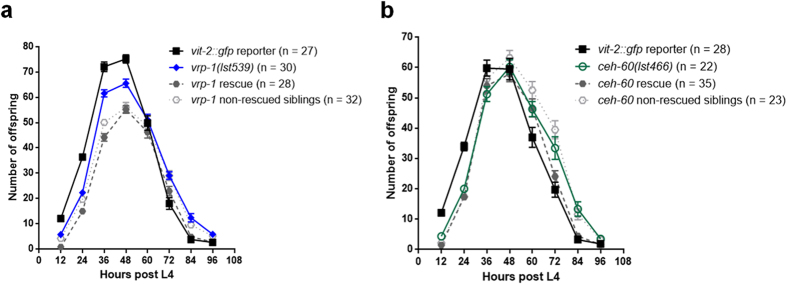
Progression of egg-laying in YPR mutants. Three-hourly collected mean values ± SEM (n = number of adults evaluated) of offspring were binned per 12 hours to create egg-laying profiles for *vit-2::gfp*, (**a**) *vrp-1(lst539)*, (**b**) *ceh-60(lst466)*, and both rescued and non-rescued siblings from extrachromosomal rescue strains of each of these mutants. Compared to *vit-2::gfp*, both *vrp-1* and *ceh-60* mutants display similar viable offspring numbers (also Supplementary Fig. S6). (**a+b**) Potential small delays in egg-laying could not be rescued.

To evaluate the effect of a strongly impaired yolk protein pool on reproductive potential, we determined the overall brood size for all YPR mutants, as well as a more detailed progression of egg-laying for *vrp-1(lst539)* and *ceh-60(lst466)* mutants, and the *vit-2::gfp* strain. *lrp-2* mutants were not considered here, since internally hatched larvae tend to damage the mother's gonad, impeding the production of eggs. Strikingly, the total amount of viable offspring is unaffected in all YPR mutants under study (Supplementary Fig. S6). In addition, while the timing of egg-laying at best seems to be slightly delayed in *vrp-1* and *ceh-60* mutants, this phenotype could not be rescued by reintroducing YPR wild-type copies capable of restoring *vit-2::gfp* expression in these strains (Fig. 5). Thus, despite their disastrous effect on endogenous yolk protein production, the studied YPR alleles do not seem to affect egg-laying.

### Survival upon starvation-induced L1 diapause

**Figure 6:**
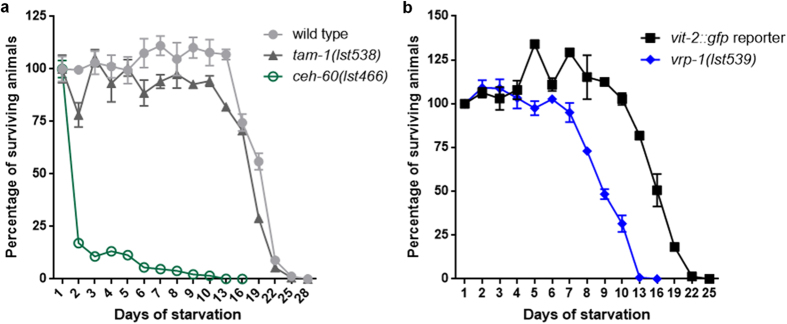
L1 diapause survival is distinctly affected in *ceh-60* and *vrp-1* mutants. (**a**) As opposed to controls, *ceh-60(lst466)* (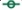) individuals cannot cope with survival in absence of food (*p* = 1.7E-09) (also Supplementary Fig. S7). While also declining slightly earlier, *tam-1(lst538)* (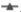) controls do not differ significantly from wild-type decline (*p* = 0.669). (**b**) Also *vrp-1(lst539)* (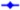) shows a decrease in fitness under these conditions (*p* = 8.68E-05), though far less outspoken than the *ceh-60(lst466)* mutant. Because L1 diapause survival depends on culture density, panels (**a**) with 11 worms/μl and (**b**) with 18 worms/μl cannot be directly compared^112^.

*vrp-1* and *ceh-60* YPR mutants cultured under standard laboratory conditions do not display overt defects in fertility nor development, despite their endogenous yolk protein pools being considerably depleted. This might be due to the presence of plentiful food in the worm’s vicinity. Yolk has been suggested to serve as an important energy source for larval survival upon L1 diapause - an alternate larval stage entered when *C. elegans* embryos hatch in the absence of food^38^,^39^. We found that deprived *ceh-60(lst466)* larvae display a convincing survival defect (Fig. 6a and Supplementary Fig. S7). At a worm density of 11 worms/μl, less than 20% of *ceh-60(lst466)* larvae survive their first day of starvation (*p* = 1.7E-09), as opposed to a median survival of 19 and 17.5 days of the wild-type and *tam-1(lst538)* controls. Though survival of L1 diapause for *tam-1(lst538)* mutants drops slightly faster than wild type, this difference is not significant (*p* = 0.669); supporting a similar behaviour for all control strains (Fig. 6a). *vrp-1(lst539)* results in a reduced survival of about 7 days compared to its control (*p* = 8.68E-05), although far more modest than that observed for *ceh-60(lst466)* (Fig. 6b). Our findings are therefore consistent with these yolk-reducing mutations having an important role for larval survival under conditions of limited food availability.

## Discussion

Invertebrate reproduction is a process that for diverse reasons grasps the attention of many. For one, egg-laying is an easily measurable phenotypic readout, exploited to raise our fundamental understanding of biological signalling pathways^40^,^41^,^42^. In addition, there’s a solid industrial interest in applying new findings in this field to diversify invertebrate pest control^43^,^44^,^45^,^46^. Most importantly, if we were to better understand the molecular mechanisms acting to control invertebrate reproduction, it would help us answer important questions about evolutionary similarities and/or discrepancies^10^. Because in egg-laying species the embryo strongly depends on maternal yolk for its development^47^,^48^,^49^, we generated a *C. elegans vit-2::gfp* reporter strain to find novel genetic modifiers of yolk protein production. This helped us to further refine the scientific view on the control of yolk production and reproduction in a nematode, revealing some unexpected insights.

While specifically looking for mutants in which the developmental fate of yolk proteins is inappropriately executed, we identified three novel genetic components involved in the *C. elegans* stage-, sex- and tissue-specific *vit* regulatory system.

The DNA-binding CEH-60 (*C. elegans* homeobox) protein is homologous to the *Drosophila melanogaster* EXD (extradenticle) protein, encoding a HOX (homeobox) cofactor^50^,^51^, and its vertebrate counterparts, the PBX (pre-B cell leukaemia transcription factor) proteins^52^. PBX1 proteins are molecular mediators of sexual differentiation^53^,^54^. The here described role for *ceh-60* in *C. elegans* yolk protein production therefore adds experimental support for a fundamental similarity between the vertebrate and invertebrate control systems of reproduction. However, its exact role within these systems might vary over species. We here confirmed previously reported expression of *ceh-60* in only a few amphid neurons implicated in sensing environmental cues^35^,^55^. These findings now set the scene for a detailed search for environmental inputs that via neuronal signalling potentially modulate intestinal function related to reproduction. In mice, PBX1 proteins act in the gonadotropin-releasing hormone neurons, which further supports the potentiality for such an axis to exist^56^.

We isolated another putative transcription factor, which we annotated as VRP-1 (vitellogenin-regulating *Caenorhabditis*-specific protein), and showed it to be an intestinal player. As such, VRP-1 is well-positioned to directly influence yolk protein production, a process that occurs in the intestine only. However, in contrast to the *vit* genes which are only expressed in adult hermaphrodites^18^, *vrp-1* is also expressed in larvae, albeit at much lower levels. These findings point to a broader role for *vrp-1*, which may from the L3 stage onwards (upon considerable up-regulation) start to serve the time- and tissue-specific regulation of *vit* expression.

To date, no (in)vertebrate orthologues of these two *C. elegans vit* regulators have been reported to be involved in yolk protein production. Moreover, all our bioinformatic searches support the *Caenorhabditis*-specific nature of VRP-1, indicating that in *C. elegans*, the regulation of yolk protein production might have at least one genus-specific aspect.

We were unable to rescue *vit-2::gfp* reporter expression of the *lrp-2(lst464)* mutant. Even though relying on tight mapping results, its annotation in our isolated strain is therefore supported by indirect evidence. Yet, the reduced *vit* transcript and yolk protein levels in the independently isolated *lrp-2(gk556842)* mutant^32^ indicate that LRP-2 is an actual yolk protein regulator. The LRP-2 (low-density lipoprotein (LDL) receptor related) protein is expressed in a multitude of tissues^57^ and is, like all known yolk receptors, a member of the LDL receptor superfamily of lipoprotein receptors^58^,^59^. Generally, such receptors are known to orchestrate (in)vertebrate cholesterol homeostasis^60^, a process that is crucial to reproduction since in most studies so far, all known regulatory pathways converge onto the production of steroid hormones. In *C. elegans*, extracellular sterols are possibly taken up by the digestive tract or through the cuticle, and are of vital importance for reproduction^5^. LRP-2 might be involved in LDL-derived cholesterol distribution and transport to hypodermal and neuronal tissues for synthesis of the endogenous sterol-derived dafachronic acids (DAs)^61^,^62^,^63^,^64^. These hormones play a role in promoting reproductive development by bypassing entry into the alternative L3/dauer larval stage^65^. If the similarity to other (in)vertebrate systems would hold, these could also be functionally involved in the control of yolk protein synthesis in the adult. In a preliminary test, we were unable to rescue any of the YPR mutant phenotypes by supplementing the worms with (25S)-Δ^7^-DA, a *C. elegans* steroid hormone able to fully rescue constitutive dauer mutants^66^. Functional relations between the YPR proteins and DA signalling nevertheless remain possible. Providing the mutants with (25S)-Δ^7^-DA alone might be an insufficient or incorrect source of steroid. Because of the tight link between vitellogenesis and steroidogenesis^1^,^2^, it will be of paramount importance to further study possible interactions and dependencies in *C. elegans*.

*lrp-2* mutants display the ‘bag of worms’ phenotype, a viviparity-like strategy. The loss of yolk production, caused by a malicious mutation in one of the regulatory genes controlling vitellogenesis, might therefore have initiated an escape mechanism, i.e. internal hatching^67^. Several other causes exist for this strategy, including deficient vulva development or inadequate motility of the vulval muscles. *lrp-2* mutants indeed suffer from insufficient yolk protein availability, but the gene is also expressed in nervous tissue and in vulval and uterine muscles. It has been described before that LRP-1 and 2 are cooperatively required for the fibroblast growth factor EGL-17 (egg-laying defective)-dependent regulation of sex myoblast migrations during larval development^68^, and as such are involved in the generation of uterine and vulval musculature^69^. For both independent *lrp-2* mutants, we were so far unable to separate their suppressed yolk protein levels from their ‘bag of worms’ phenotype. Taken together, these findings point towards a tight link between the EGL circuit and yolk production. This is further supported by compromised egg-yolk pools in *egl-15* knockdown animals, as observed by others^25^.

Vitellogenesis encompasses an important metabolic cost and the number of genes involved in the production of *vit* mRNAs may be surprisingly large^3^. It can therefore be asked whether and how the novel genetic players retrieved from our screen may act together to influence *C. elegans* yolk protein production. Since we were mainly interested in the control of yolk protein synthesis, we did not specifically survey the involvement of yolk and yolk receptor endocytic trafficking components in this regard. Nevertheless, it is possible that transcriptional *vit* regulators also affect the level of yolk transport, e.g. through control of the earlier described factors RME-2, RAB-35, RAB-10 and M04F3.2^6^,^24^,^25^.

Based on the transcriptional and translational data, some initial mechanistic concepts were obtained. Overall, the YP170::GFP fusion protein pool is more prone to suppression by the here identified yolk protein regulators than the endogenous YP170 yolk protein pool in the reporter background. However, also in a wild-type background - i.e. having only the endogenous yolk protein pool - YP170 levels are severely reduced for YPR mutant alleles. The *vit-2::gfp* reporter construct probably acts as a sink for transcription factors and their potential regulators.

Regarding LRP-2, the RNA data were not unambiguous since we detected wild-type levels of *lrp-2* transcripts in the *lrp-2* mutant. Due to its large size (~14.5 kbp), the *lrp-2* transcript might be less vulnerable to nonsense-mediated mRNA decay^70^,^71^,^72^, resulting in a slower turnover and possibly explaining the higher detection levels. Furthermore, another study classified L3-to-adult mRNA profiles of thousands of transcripts as either “flat”, “rising” or “oscillating”^73^. Supporting our observations, all *vit* genes were classified as rising, *vrp-1* as rising and *ceh-60* as oscillating. Contrary to our data, *lrp-2* was classified as flat. While these authors measured expression levels at 25 °C and therefore may have started their observations right after the here observed *lrp-2* peak, this discrepancy should caution against conclusions solely based on the *lrp-2* expression profile. Yet, behaving quite different from *vrp-1* and *ceh-60*, it seems that the LRP-2 receptor regulates yolk protein production distinctly from the proposed signalling interactions described below.

Our data support that *ceh-60* and *vrp-1* influence each other’s expression levels, arguing for a signalling system between the amphids and the intestine characterized by bidirectional information transfer. CEH-60 is well-localized to integrate environmental information important for egg maturation. Based on its nuclear localization and bioinformatic predictions, VRP-1 may well be a transcription factor. *vrp-1* expression starts to rise prior to the marked *ceh-60* boost (the latter upon completion of larval development), but without this boost, its expression levels cannot rise any further. Conversely, without proper *vrp-1* expression, *ceh-60* levels remain low as of L4.

It has been shown that *ceh-60* and all *vit* genes are down-regulated in egg-laying defective *ets-4* mutants^74^. This suggests that the intestinal longevity player ETS-4 communicates with CEH-60 to integrate longevity cues in the system, which makes sense from an energetic point of view: environmental cues should generally coincide with other factors, e.g. information on nutrients available via the intestine. In *D. melanogaster* a mechanism has been described wherein the presence of dietary compounds in the intestine enhances, via intermediate player(s) yet to be identified, yolk protein gene expression in the fat body and thus egg production^9^. The initiation of vitellogenesis in *Aedes aegypti* and *Sarcophaga bullata* in response to, respectively, a blood or liver meal, is yet another well-known indicator of the existence of such a control mechanism^7^,^75^,^76^,^77^. In *C. elegans*, other previously identified intestinal *vit* regulators, such as the lipid metabolism-regulating transcription factor KLF-3 (Krüppel-like factor (zinc finger protein))^78^,^79^ and to a lesser extent RBPL-1 (retinoblastoma binding protein like)^80^, are also possibly involved in the integration of signals regarding the nutritional status of the intestine, i.e. the prime site of lipid metabolism^81^. Future experiments will be needed to reveal the interplay of these regulators with the here identified regulators of yolk protein production. Particularly KLF-3 is of interest, since the vertebrate CEH-60 homolog PBX1 is known to modulate transcriptional regulation via the KLF-3 homolog, KLF4^82^.

The view that energetically costly investments in the production of egg-yolk are needed to enable viable offspring is - in nematodes - supported by the phenotype of *rme-2* mutants. In these mutants, the slightly smaller oocytes lack yolk proteins due to a malfunctioning of yolk endocytosis, ovulation is defective and both the production of embryos as well as their viability is reduced^6^. While it should be noted that our particular forward genetic approach restricted the identification of yolk protein regulatory mutants to those able to reach fertile adulthood, it should still enable the isolation of mutants with a severely reduced reproduction potential. Therefore, we wanted to assess whether for the *vrp-1* and *ceh-60* mutants, yolk protein production would indeed be a predictive criterion for overall reproductive success.

Contrary to what would be assumed based on the abundant expression of six *vit* genes in wild-type adults^36^, YPR mutants displayed no clear reproduction defects with the exception of the ‘bag of worms’ phenotype for *lrp-2* mutants. Indeed, strongly reduced yolk protein pools are not necessarily critical for successful brood development in our *C*. *elegans* experiments, as opposed to e.g. *D. melanogaster*^83^. The simplest explanation would be that in these mutants, the little remaining fuel suffices for the production of the observed amount of eggs. This could suggest that the enormous amounts of yolk serve another purpose.

Even though hermaphrodites usually self-fertilize in the wild^84^, they might *de facto* prepare for the higher offspring numbers observed when mated with a male^85^,^86^. However, we observed far greater reductions in yolk protein content in our set of mutants than can directly be correlated to this phenomenon. Alternatively, yolk proteins could hypothetically be implicated in yet unidentified, co- and post-reproductive processes in the adult^87^. This is somewhat supported by the observation that yolk synthesis, once induced, is not switched off again in post-reproductive adults, although this might equally well be an unwanted side-effect inherent to ageing, as explained by Blagosklonny’s hyperfunction theory^88^,^89^.

These two possibilities are nevertheless difficult to reconcile from an evolutionary point of view: why would nature have specifically selected those *C. elegans* that invest in immense amounts of yolk production, if it hadn’t provided them with a general hermaphrodite-specific advantage up to their reproductive period? We therefore timed the egg-laying profile of selected YPR mutants with great detail. While their overall offspring numbers are nearly similar to wild-type levels, *vrp-1* and *ceh-60* mutants displayed a small delay in egg-laying. In the wild, where resources are scarce, the massive *C. elegans* yolk protein supplies may therefore capacitate the fertile adult with a very efficient way of outcompeting others. We could however not rescue this phenotype, arguing against this hypothesis. Alternatively, it could be possible that levels capable of restoring *vit-2::gfp* reporter expression may yet not suffice to restore other phenotypical consequences. This can be understood from the clear preference of VIT-2::GFP production over endogenous yolk in the reporter strain. Taken together, this means that *vrp-1* and *ceh-60* genes at best have a mild influence on the timing of egg-laying under standard laboratory conditions. However, in the wild, these ample-food conditions are presumably rarely met.

Therefore, we attempted to demonstrate the importance of yolk for larval survival during L1 diapause emergence in YPR mutants^39^. When hatched in the absence of food, the survival of *vrp-1(lst539)* and *ceh-60(lst466)* yolk-depleted L1 larvae is moderately to severely compromised, respectively. These findings at least suggest that while the remaining low levels of yolk in these mutants may suffice under rich nutritional conditions, they represent a disadvantage when food is in short supply. In the adult, decisions with respect to entering the reproductive state depend on the environment’s nutritional value and are taken long before *ceh-60* and *vrp-1* can act on adult *vit* expression (i.e. the L3-L4 moult). The observed abundance of yolk under optimal conditions must therefore at least in part be a consequence of the natural selection events in favour of high yolk amounts to preserve L1 survival in the absence of food.

Our data on reproduction might also comply with a more refined escape mechanism, in which possible compensating substances preserve embryo viability - thus reproductive success - but not necessarily L1 diapause survival when yolk protein levels are low. After fertilization and cleavage, the yolk particles of control animals are approximately evenly distributed among the daughter cells and only slowly metabolized^6^. Considerable amounts of yolk remain in newly hatched larvae, the majority of which in intestinal cells. They continue to utilize yolk as a food source, reflected by the fast degradation of residual yolk proteins in L1 larvae. Corresponding phenomena are observed in amphibians^90^ and insects^91^,^92^. This observation has led to the assumption that yolk proteins must initially be present in excess^19^, but in fact, it could even be true that in *C. elegans*, embryonic development does not strongly rely on yolk. Alternative nutrients could be transported into the embryo, a process that could involve a more general transport of lipoproteins via the *C. elegans* yolk receptor, RME-2^6^, in addition to yet unknown transport mechanisms. If these substances are inadequate to serve as backup under harsh conditions, this would still be in line with the observed L1 diapause defects.

In conclusion, starting from a forward genetic screen, we could identify novel genetic regulators of yolk protein production in *C. elegans*, hereby improving our knowledge on invertebrate control of yolk production, a process assumed to only serve reproduction. Our data support that parallel pathways are active in multiple tissues, and that the proteins VRP-1, CEH-60 and LRP-2 are involved in the general regulation of *C. elegans* yolk protein production. Based on our data, it seems that *C. elegans* produces far more yolk than is needed to sustain its embryos. Enormous amounts of yolk might have been selected for in the wild, enabling not embryonic, but larval survival when resources are scarce. Yet, our findings still suggest an additional investment in yolk, opening up new research possibilities as to the why and how of this energy-costly process.

## Methods

### Strains, microscopy and growth conditions

Following strains were obtained via the CGC: **N2** *Bristol*^93^ wild-type control, **CB4856** *Hawaiian*^94^ isolate and **VC40291** lrp-2(gk556942) null mutant^32^. The **UL2612** *Pceh-60::gfp* strain was a kind gift of Professor I. A. Hope, University of Leeds, Leeds, UK^35^. For the forward genetic screen, we generated **LSC276** *lstIs13 [Pvit-2::vit-2::gfp]* as starting strain to avoid potential non-standard *unc-119* expression levels in the existing RT130 *unc-199(ed3); pwIs23 [Pvit-2::vit-2::gfp, unc119(+)]* strain. LSC276 was then used to obtain the following mutant strains: **LSC462** *rab-35(lst462); lstIs13 [Pvit-2::vit-2::gfp]*, **LSC463** *rab-10(lst463); lstIs13 [Pvit-2::vit-2::gfp]*, **LSC537** *M04F3.2(gk2294); lstIs13 [Pvit-2::vit-2::gfp]*, **LSC648** *vrp-1(lst461); lstIs13 [Pvit-2::vit-2::gfp]*, **LSC649** *ceh-60(lst491); lstIs13 [Pvit-2::vit-2::gfp]*, **LSC650** *ceh-60(lst466); lstIs13 [Pvit-2::vit-2::gfp]*, **LSC651** *vrp-1(lst539); lstIs13 [Pvit-2::vit-2::gfp]*, **LSC652** *tam-1(lst538); lstIs13 [Pvit-2::vit-2::gfp]* and **LSC653** *lrp-2(lst464); lstIs13 [Pvit-2::vit-2::gfp]*. Mutant strains were backcrossed several times (Table 1) with LSC276 (*lstIs13 [Pvit-2::vit-2::gfp]*) males on the basis of their aberrant yolk protein synthesis phenotype, and maintained as homozygotes according to standard methods^93^. Similarly, the lrp-2(gk556942) mutant was backcrossed twice to N2 males based on its ‘bag of worms’ phenotype. We furthermore outcrossed *vrp-1(lst539); lstIs13 [Pvit-2::vit-2::gfp]*, *ceh-60(lst466); lstIs13 [Pvit-2::vit-2::gfp]*, *ceh-60(lst491); lstIs13 [Pvit-2::vit-2::gfp]* and *lrp-2(lst464); lstIs13 [Pvit-2::vit-2::gfp]* animals to N2 in order to remove the transgenic array from its background and respectively obtained strains **LSC901** *vrp-1(lst539)*, **LSC897** *ceh-60(lst466)*, **LSC903** *ceh-60(lst491)* and **LSC904** *lrp-2(lst464)*. The causal allele of each (back- or outcrossed) mutant used during further analyses was confirmed by PCR and Sanger sequencing (Supplementary Table S4). For all these analyses, random animals from the population were always used.

All fluorescence and bright-field micrographs in this study were obtained with an Axio Imager. Z1 light microscope equipped with an AxioCam MRm camera (1388 × 1040 pixels) using the digital image processing software ZEN 2012 (Carl Zeiss Microscopy, Göttingen, Germany) and identical settings.

Amphid neurons of *Pceh-60::gfp* worms were stained with 1,1'-dioctadecyl-3,3,3',3'-tetramethylindocarbocyanine perchlorate (DiI; Molecular Probes, Carlsbad, California)^95^,^96^,^97^. Fluorescent signals were visualized by an Olympus Fluoview FV1000 (IX81) confocal microscope, and confocal Z-stack projections were exported through Imaris 7.2 (Olympus, Tokyo, Japan).

All strains were maintained at 20 °C on standard nematode growth medium (NGM) agar plates seeded with *Escherichia coli* OP50^98^.

### Transgenic *C. elegans* strains

#### Vitellogenin reporter

We constructed a transgenic vitellogenin reporter strain for a subsequent forward genetic screen. The plasmid V2B3 (a kind gift of Professor B. Grant, Rutgers University, New Jersey), encoding the full-length yolk protein YP170B fused to GFP^99^ and expressed under *vit-2* promoter control, was injected at 80 ng/μl into N2 worms using standard microinjection techniques^100^. One stably integrated line was produced using a UV crosslinker (BLX-254; Vilber Lourmat, Marne-la-Vallée, France) following standard protocols and backcrossed ten times to N2 to obtain the *lstIs13 [vit-2::gfp]* reporter strain^101^,^102^,^103^.

#### YPR mutant rescues

For *vrp-1(lst539)* and *ceh-60(lst466)* strains, gDNA rescue experiments by germline transformation were executed. We PCR amplified gDNA rescue fragments using the *Pfu* DNA polymerase (Thermo Scientific, St. Leon-Rot, Germany) and oligonucleotides listed in Supplementary Table S5. These fragments contain the full-length gDNA, the upstream promoter and downstream regulatory sequence. Rescue strains were obtained from microinjections at 20 ng/μl for *ceh-60(lst466)* (**LSC898**) and 50 ng/μl for *vrp-1(lst539)* (**LSC899**) with an intestinal *Pelt-2::mCherry* selectable co-injection marker.

Similarly, microinjections at 5, 20 and/or 50 ng/μl were carried out in order to obtain the genomic rescues of the non-backcrossed mutant strains (Supplementary Fig. S1).

A rescue analysis using *vrp-1* cDNA sequence was additionally carried out. Overlapping fragments of the *vrp-1* promoter region (306 bp) and *vrp-1* cDNA were generated using the primer sets listed in Supplementary Table S5, and subsequently fused using a PCR fusion-approach^104^. The obtained fragment was fused to an overlapping fragment of the 3′ UTR of *vrp-1* and microinjected at 50 ng/μl.

#### VRP-1 reporter strain

For *in vivo* localization of VRP-1, overlapping fragments of full-length *vrp-1* gDNA lacking its stop codon together with a 342 bp upstream regulatory region and the *gfp* region of pPD95.75 (Fire Lab *C. elegans* Vector Kit, 1995) were first amplified by PCR (oligonucleotides: see Supplementary Table S6). Subsequently, these fragments were fused with a set of nested primers to obtain a translational *Pvrp-1::vrp-1::gfp* fusion construct driven by a promoter region of 306 bp^104^. Several independent transgenic lines were obtained by germline transformation of N2 worms with 50 ng/μl of *Pvrp-1::vrp-1::gfp* co-injected with a pan-neuronal *Prgef-1::rfp* selectable marker (contained on plasmid pCB101, a kind gift of Professor M. Doitsidou and Professor O. Hobert, Colombia University, New York)^105^.

### Yolk protein mutant isolation

For the manual screen, early L4 *lstIs13 [Pvit-2::vit-2::gfp]* animals were mutagenized with 50 mM of EMS according to standard protocol^93^. After 24 hours, 3 to 5 mutagenized gravid adults were placed in each of a total of 40 founder P0 plates. During the next 12 hours, all F1 progeny (~3,750) of the mutagenized P0 animals were singled out. *gfp* expression from the *lstIs13* transgene of the ensuing progeny (F2 generation) was scored via microscopic observation (Leica MZ16 F; Leica Microsystems, Wetzlar, Germany). Individual mutants were picked and their phenotypic heritability confirmed in order to establish mutant lines. By first crossing mutant worms with both N2 and premutagenized *lstIs13* males and then assessing fluorescence levels, we determined whether mutations were dominant or recessive, or alternatively, whether the *gfp* array was hit.

### Sequencing of YPR mutants

#### WGS SNP mapping strategy

Following the guidelines previously described^22^, we set up crosses between ten of the isolated, homozygous recessive mutant strains (*Bristol* background) and CB4856 *Hawaiian* males. ~50 F2 recombinant worms (Supplementary Fig. S1) segregating from this cross and displaying the proper mutant phenotype were individually reselected. They were allowed to self-fertilize and their F3 and F4 progeny were pooled. For mutants displaying complete loss of *gfp* expression, only plates originating from F2 animals that appeared to be positive (but were not particularly homozygous) for the *gfp* reporter gene, as assessed by PCR, were pooled (oligonucleotides: Supplementary Table S4). Genomic *C. elegans* DNA samples were prepared^22^ and at least 3 μg of genomic DNA (gDNA) (≥30 ng/μl) from each sample was submitted to BGI Tech Solutions Co., Limited (Hong Kong, China) for short-insert library preparation and 100 bp paired-end sequencing on an Illumina HiSeq 2000 sequencer to obtain 3 Gb clean data, corresponding to a ~30-fold genome coverage across all non-gap regions. A gDNA sample of pure background strain was similarly handled to obtain 1 Gb clean data.

#### WGS data analysis using the CloudMap pipeline

The generated Illumina 1.5 FASTQ data files were uploaded into Galaxy and analysed using the CloudMap *Hawaiian* variant mapping with WGS and variant calling workflow (http://usegalaxy.org/cloudmap)^106^. Two pre-processing steps were run on the FASTQ files in Galaxy. First, we concatenated the FASTQs for each sample using the Concatenate Datasets tool and subsequently converted the resulting file into FASTQ Sanger quality encoding using the FASTQ Groomer tool^107^. Prior to the automated execution of the different implicated bioinformatics processing steps, we adapted the workflow to accept paired-end FASTQ files according to the user guide. The default tool settings further used have thoroughly been described before^106^.

### Yolk protein analysis

The YP170 yolk protein pool was analysed for each condition as previously described^33^, with animals growing on plates (90 mm diameter) treated topically with 80 μl of 50 mM 2′-deoxy-5-fluorouridine (FUdR; Sigma-Aldrich, St. Louis, Missouri) to avoid offspring accumulation^108^. This did not result in visible changes of *vit-2::gfp* reporter expression of the control worms. Since yolk proteins are first expressed during L4 lethargus^18^, age-matched L4 stage larvae were obtained for each condition and 50 hermaphrodites were harvested in up to quadruplicate 24, 36, 48, 60, 75, 84 and 108 hours later into 15 μl M9 buffer, diluted in 15 μl 2x Laemmli Sample Buffer (Bio-Rad, Hercules, California) containing β-mercaptoethanol (Sigma-Aldrich). Worm samples were subsequently incubated during 15 min in a 70 °C water bath and vortexed every 5 min before optional storage at −80 °C. Prior to loading 15 μl on a 4–12% Bis-Tris Criterion XT precast polyacrylamide gel (Bio-Rad), protein samples were spun for 5 min to remove insoluble precipitates. According to the manufacturer’s instructions, gels were stained and destained using SimplyBlue SafeStain (Life Technologies).

To ultimately verify the identity of the Coomassie G-250-stained YP170 (~170 kDa) and YP170B::GFP (~180 kDa) protein bands, they were first manually excised using a fresh scalpel for each band and destained during 30 min in 100 μl 100 mM ammonium bicarbonate, interrupted by occasional vortexing. Next, we followed our general lab protocol for trypsin digestion^109^. The tryptic peptide samples were dried using a SpeedVac Concentrator (Savant) and subsequently purified to remove interfering salt contaminants using Pierce C18 Spin Columns corresponding to manufacturer’s guidelines (Thermo Scientific). The samples were dried again in the vacuum evaporator and stored at −80 °C before mass spectrometric (MS) analysis performed on an electrospray ionization quadrupole time-of-flight (ESI-QTOF) mass spectrometer micrOTOF-Q (Bruker Daltonics, Bremen, Germany) operated in reflectron positive ion mode. We identified proteins through peptide mass fingerprinting using Mascot (Matrix Science, London, UK). An error window on experimental peptide mass values of 400 ppm and on MS/MS fragment ion mass values of 600 mmu was used in all searches and we allowed one missed cleavage per peptide. Carbamidomethylation (C) and oxidation (M) were respectively chosen as fixed and variable modifications. The ~170 kDa searches were limited to *C. elegans* proteins and the ~180 kDa searches to all entries in the SwissProt database.

Protein bands corresponding to YP170 were quantified relative to myosin protein levels (~200 kDa) by using the Image Lab Software Version 4.1 (Bio-Rad).

In parallel, rat polyclonal antiserum anti-YP88 (a kind gift of Professor T. Blumenthal, University of Colorado Boulder, Colorado and Professor S. Strome, University of California, Santa Cruz, California)^3^, were applied to detect the YP88 yolk pool during immunoblot assays. Thereto, 15 μl of single fertile hermaphrodite protein samples prepared 36, 60, 84 and 108 hours post the L4 stage were separated on a 4–12% Bis-Tris Criterion XT precast polyacrylamide gel. Polyclonal rabbit anti-rat immunoglobulins conjugated to horseradish peroxidase acted as the secondary antibody (Dako, Glostrup, Denmark). Signal quantification relative to an Amersham Deep Purple total protein stain (GE Healthcare, Piscataway, New Jersey) was performed using the Image Lab Software Version 4.1.

### Real-time PCR analysis

Circa three fully-grown plates (90 mm diameter) containing age-matched populations of fertile hermaphrodite adults were obtained in up to triplicate for each condition. For developmental analysis, a time series consisting of two to three fully-grown plates per sample and per condition was collected; for the adult *vit* expression profiles, cultures were treated with FUdR as described above. Total RNA was prepared using the RNeasy kit (Qiagen, Hilden, Germany) and subjected to DNaseI (Qiagen) treatment. Next, cDNA was synthesized in duplo from up to 500 ng of total RNA in a 100 μl-volume reaction using the PrimeScript RT Master Mix (Takara, Ohtsu, Japan), upon which technical replicates were pooled. Per biological replica, technical duplicate or triplicate 20 μl qRT-PCR reactions were set up in 96 well plates using the SYBR Green PCR Master Mix (Applied Biosystems, Foster City, California), and reactions were run on the StepOnePlus Real-Time PCR System (Applied Biosystems). The primer sets used in these reactions for transcripts *vit*-*2*, *-3*, *-4*, *-5*, *-6*, *vrp-1*, *lrp-2*, *ceh-60* and *lin-42* are listed in Supplementary Table S7. The relative expression level of each gene transcript in control versus mutant was assessed by a geNorm-based normalization strategy, in which *cdc-42*, *pmp-3* and *tba-1* emerged as optimal reference genes for this study^110^. Analysis of variance (ANOVA) statistics running post hoc Dunnett’s tests were used to obtain *p*-values of significance for the analysis of individual transcripts in each mutant condition.

### Egg-laying

The progression of egg-laying and the overall brood size were determined by selecting synchronized L4 nematodes and placing them each on a single NGM plate with OP50 bacteria. From 18 hours until 96 hours after mid-L4, worms were repeatedly transferred every 3 hours to a freshly seeded NGM plate for a period of 12 hours, for practical reasons alternated with a single transfer after a full period of 12 hours. We counted the number of offspring when in the L4/adult stage. Incomplete offspring data due to escaping or dying mothers were omitted from the analyses. Total brood sizes were compared using ANOVA statistics running a post hoc Dunnett’s test.

### L1 diapause survival assay

Starvation-induced L1 diapause survival assays were performed essentially as described previously using comparable densities for mutant and control strains^39^. Synchronized L1 larvae were incubated in 3 ml of sterilized S-basal buffer at 20 °C. For each condition, aliquots containing roughly 200 animals were placed in triplicate on individual seeded plates every single day during a period of 10 days starting at the first starvation day, followed by every 3 days for the consecutive period. The number of surviving animals was counted when in the L4/adult stage at 20 °C. The number of surviving animals at the first day of starvation was set at 100% in order to calculate the percentage of surviving animals at the following time points. The L1 diapause data were statistically compared using an analysis of covariance (ANCOVA) model. The rate of survival was log-transformed to accommodate the assumption of normality, and data were analysed using *R* (The *R* Foundation for Statistical Computing, Vienna, Austria).

In general, statistical analyses and graphing in this manuscript were performed using Prism 6 (GraphPad Software, La Jolla, California), except for Fig. 3, S3 and S4 (made using Excel 2007).

## Additional Information

**How to cite this article**: Rompay, L. V. *et al.* New genetic regulators question relevance of abundant yolk protein production in *C. elegans*. *Sci. Rep.* **5**, 16381; doi: 10.1038/srep16381 (2015).

## Supplementary Material

Supplementary Information

Supplementary Table S1

Supplementary Table S2

## Figures and Tables

**Table 1:** Details of all homozygous mutant strains affecting VIT-2::GFP reporter expression.

Strain	Gene	Allelic Variant	Backcrossed	Phenotypic VIT-2::GFP category^a^
LSC462	*rab-35*	*lst462*, G121A	0x	Abnormal accumulation, fully penetrant
LSC463	*rab-10*	*lst463*, W63amber	0x	Abnormal accumulation, fully penetrant
LSC537^b^	*M04F3.2*	*gk2294*, E63ochre	0x	Abnormal accumulation, fully penetrant
LSC652	*tam-1*	*lst538*, C185Y	4x	Reduced, fully penetrant
LSC648	*vrp-1*	*lst461*, Q77ochre and Q79ochre (by alternative splicing)	6x	Complete loss, fully penetrant
LSC651	*vrp-1*	*lst539*, G1147A	4x	Incomplete loss, fully penetrant
LSC650	*ceh-60*	*lst466*, Q241ochre	4x	Incomplete loss, fully penetrant
LSC649	*ceh-60*	*lst491*, G2431A	4x	Incomplete loss, fully penetrant
LSC653^c^	*lrp-2*	*lst464*, C3875opal	2x	Incomplete loss, fully penetrant

^a^Respectively <20%, <15%, <5% and <50% of the *vrp-1(lst539)*, *ceh-60(lst466)*, *ceh-60(lst491)* and *lrp-2(lst464)* mutant populations display incomplete *vit-2-gfp* expression, unlike their siblings, which display complete loss.

^b^We isolated the exact same *gk2294* allele as earlier isolated^113^.

^c^The obvious ‘bag of worms’ phenotype is also observed in the independently isolated, twice backcrossed *lrp-2* mutant VC40291 (*gk556942*, R750opal)^32^.
