# Supplementary material for: New genetic regulators question relevance of abundant yolk protein production in C. elegans
Source: Sci Rep. 2015 Nov 10;5:16381. doi: 10.1038/srep16381 (PMC4639837; doi:10.1038/srep16381)
Supplement: Supplementary Information [file srep16381-s1.doc]

**Supplementary Information**

**New genetic regulators question relevance of abundant yolk protein production in *C. elegans***

**Liesbeth Van Rompay, Charline Borghgraef, Isabel Beets, Jelle Caers and**

**Liesbet Temmerman***

Functional Genomics and Proteomics

Department of Biology, KU Leuven

3000 Leuven, Belgium

*Corresponding author: Liesbet.Temmerman@bio.kuleuven.be

**Content**

**Supplementary Figures S1-7**

- Figure S1: a-b p4 ; c-d p5; legend p6
- Figure S2: a p7, b-c p8, d-e p9; legend p10
- Figure S3: a p11, b p12, c p13, d p14, legend p14-15
- Figure S4: p16
- Figure S5: p17
- Figure S6: p18
- Figure S7: p19

**Supplementary Tables S1-7**

- Table S1: legend p21, table metadata
- Table S2: legend p21, table metadata
- Table S3: p21
- Table S4: p22
- Table S5: p23
- Table S6: p24
- Table S7: p24

**Supplementary References: p25**

**Supplementary Figures S1-7**

**New genetic regulators question relevance of abundant yolk protein production in *C. elegans***

**Liesbeth Van Rompay, Charline Borghgraef, Isabel Beets, Jelle Caers and**

**Liesbet Temmerman***

Functional Genomics and Proteomics

Department of Biology, KU Leuven

3000 Leuven, Belgium

*Corresponding author: Liesbet.Temmerman@bio.kuleuven.be

**Figure S1 (a-b)**

**
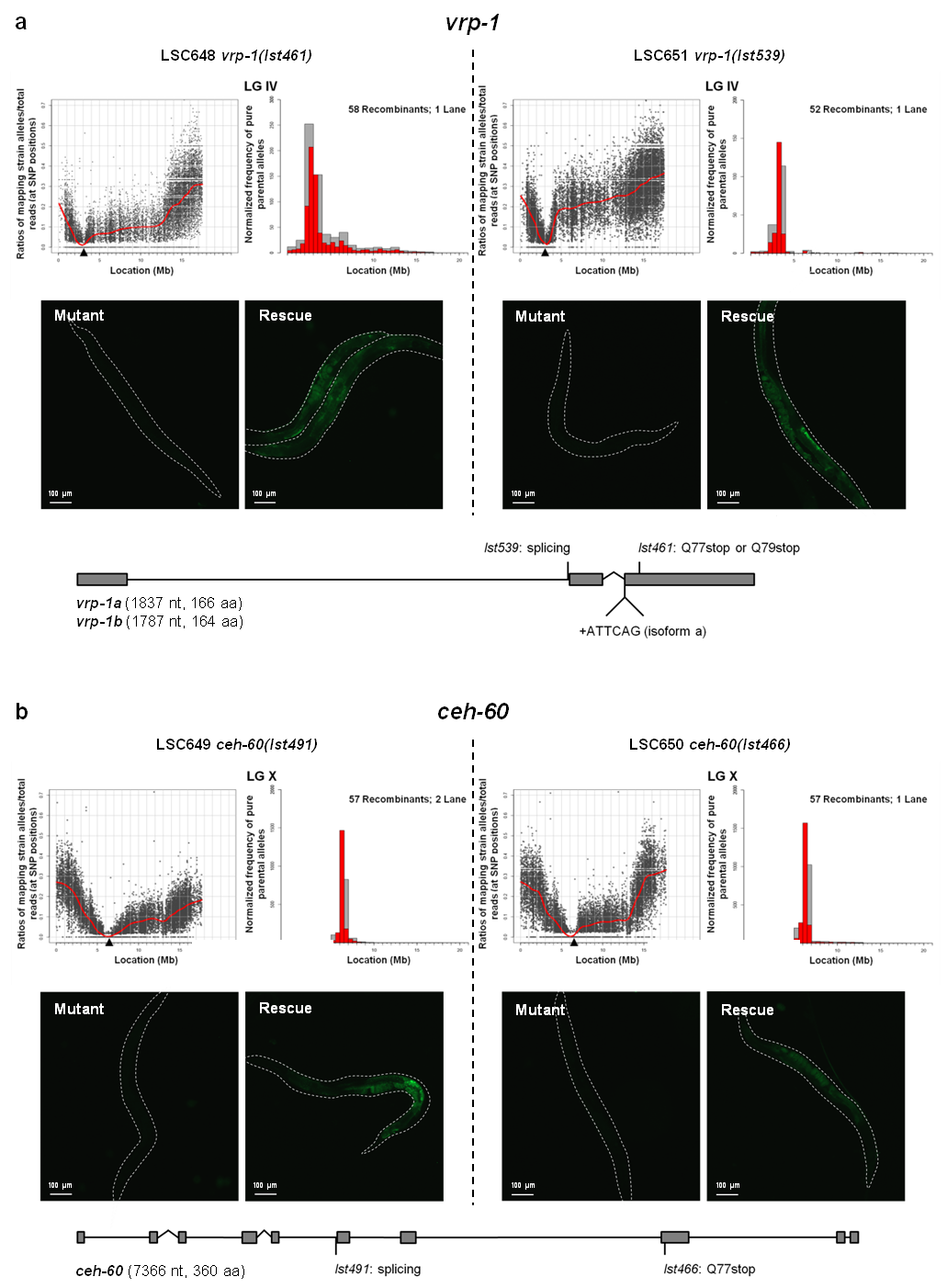
**

**Figure S1 (c-d)**

**
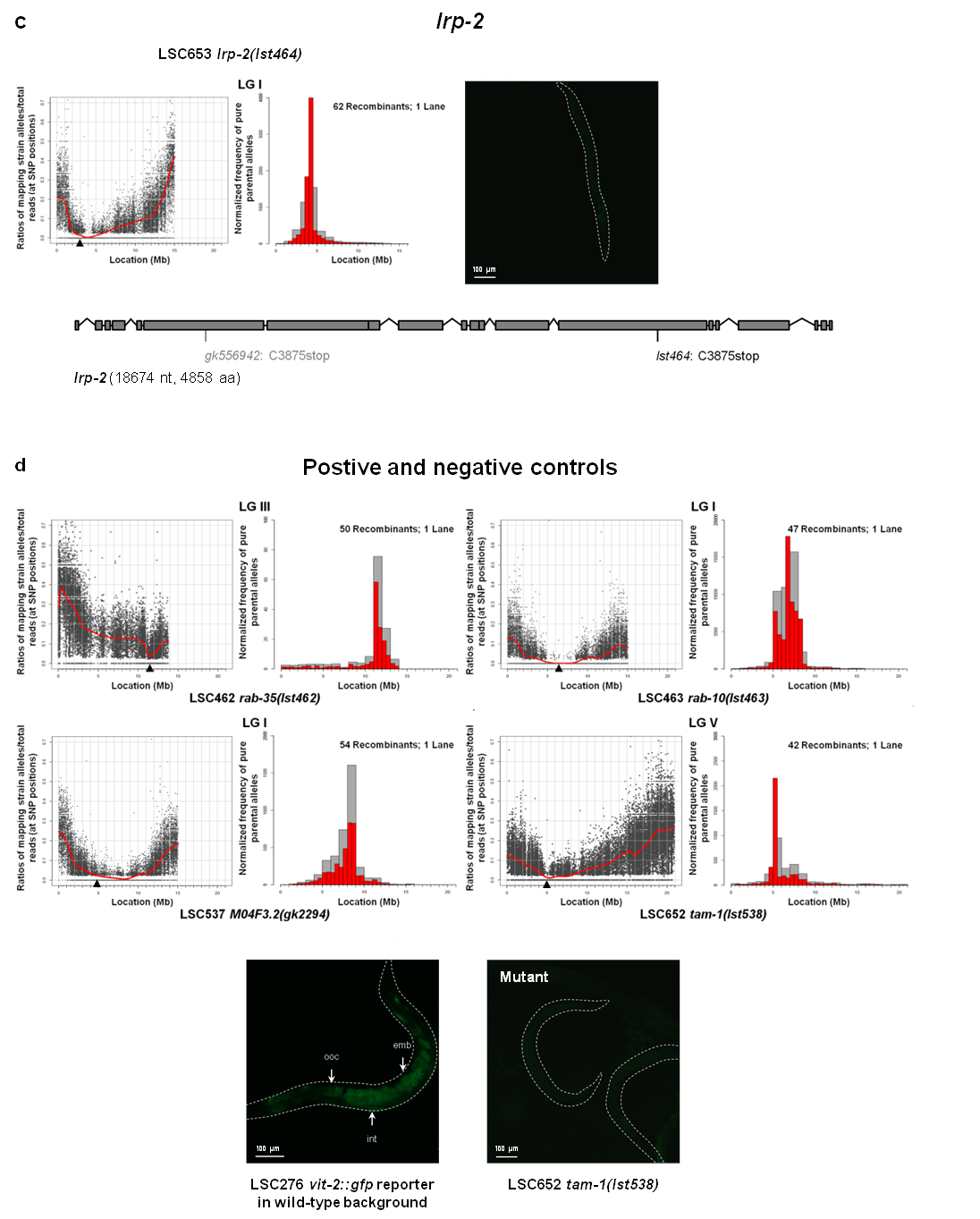
**

**Figure S1. Positional cloning of VIT-2::GFP regulating genes.**

Top panels summarize the WGS SNP mapping results for **(a)** *vrp-1(lst461)* and *vrp-1(lst539)*, **(b)** *ceh-60(lst491)* and *ceh-60(lst466)*, **(c)** *lrp-2(lst464)* and **(d)** *rab-35(lst462)*, *rab-10(lst463)* and *M04F3.2(lst537)* mutants, and the negative *tam-1(lst538)* mutant control. For each selected mutant, the XY scatter plot representing the ratio of *Hawaiian* reads/total read depth for each SNP position is shown only for the linkage group (LG) carrying the phenotype-causing variant (arrowhead). A cluster of data points dropping to y = 0 typifies the specific linked genomic region and is more explicitly visualized by a LOESS regression line (red) plotted through all data points. Accompanying frequency plots of pure parental allele SNP positions display a matching peak in the same region and are grouped into 1 Mb (grey) and 0.5 Mb (red) bins. Numbers of exact pooled recombinants and used sequencing lanes for data analysis are indicated. Middle panels display VIT-2(YP170B)::GFP reporter phenotype of the **(a)** *vrp-1(lst461)* and *vrp-1(lst539)*, **(b)** *ceh-60(lst491)* and *ceh-60(lst466)* mutants (left) and corresponding genomic rescues (right), the **(c)** *lrp-2(lst464)* mutant, and **(d)** the negative *tam-1(lst538)* mutant and *vit-2::gfp* reporter controls. Fluorescence from the YP170B yolk protein reporter in a wild-type adult hermaphrodite can be observed in the intestine (int), late-stage oocytes (ooc) and embryos (emb). Fluorescence micrographs of representative animals illustrate that introduction of the appropriate genomic transgene fully recues *gfp* expression. Respectively < 20%, < 15%, < 5% and < 50% of the *vrp-1(lst539)*, *ceh-60(lst466)*, *ceh-60(lst491)* and *lrp-2(lst464)* mutant populations display incomplete *vit-2-gfp* expression, unlike their siblings, which display complete loss. For *vrp-1(lst461)* and *tam-1(lst538)* mutants a respectively complete loss of *vit-2::gfp* expression and a less intense (i.e. reduced), yet normal *vit-2::gfp* expression pattern could be observed for all individuals. LSC numbers refer to strain names. Bottom panels display gene structures for **(a)** *vrp-1*, **(b)** *ceh-60* and **(c)** *lrp-2* (not to the same scale, adapted from WormBase). Physical locations of the novel YPR mutant alleles originating from this study are indicated in black and the previously described *lrp-2(gk556942)* variant ^1^ is indicated in grey.

**Figure S2a**


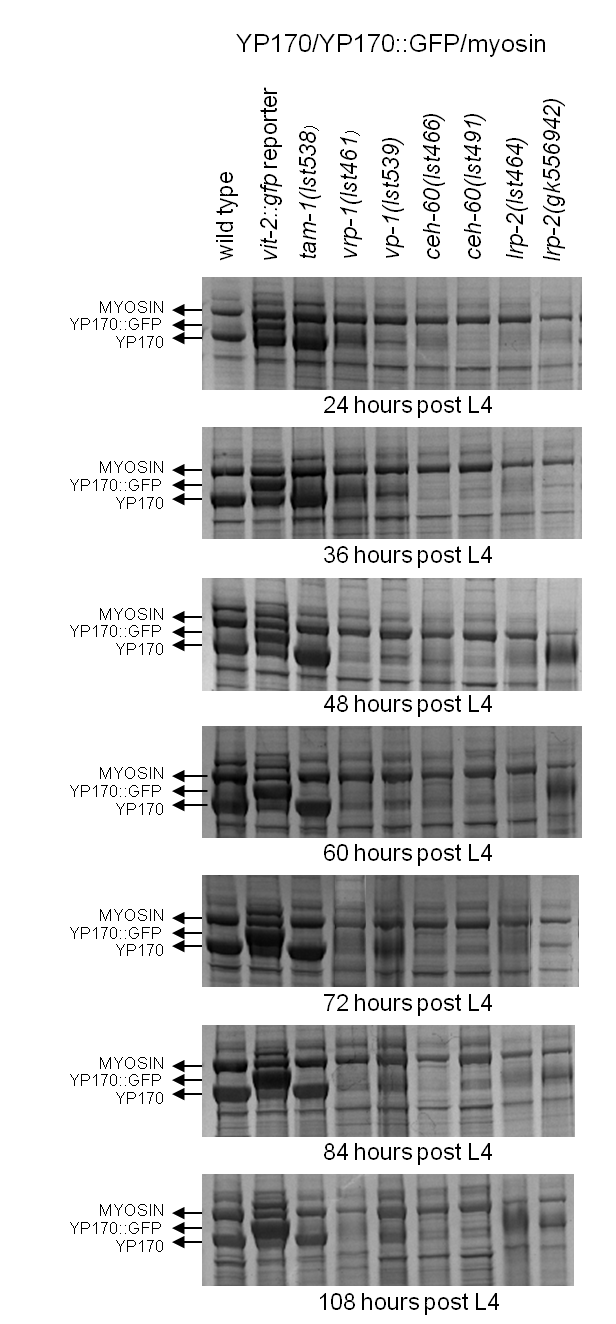

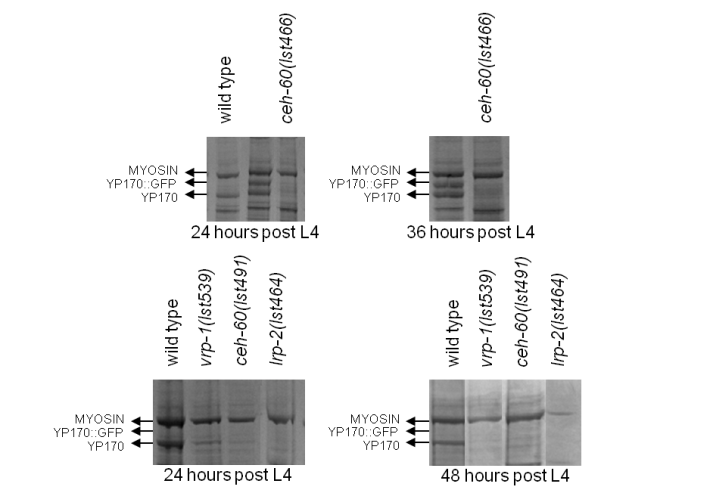


**Figure S2b**

**
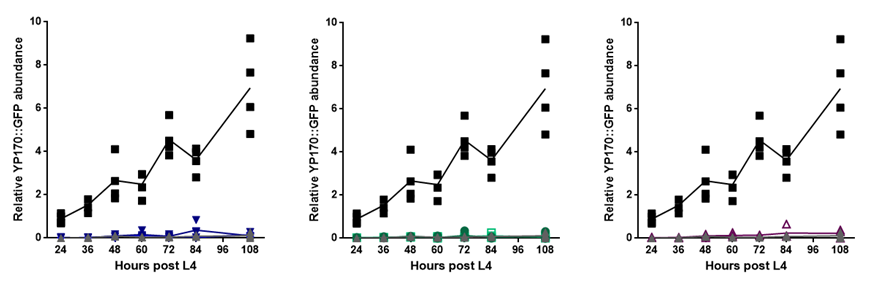
**

**Figure S2c**

**
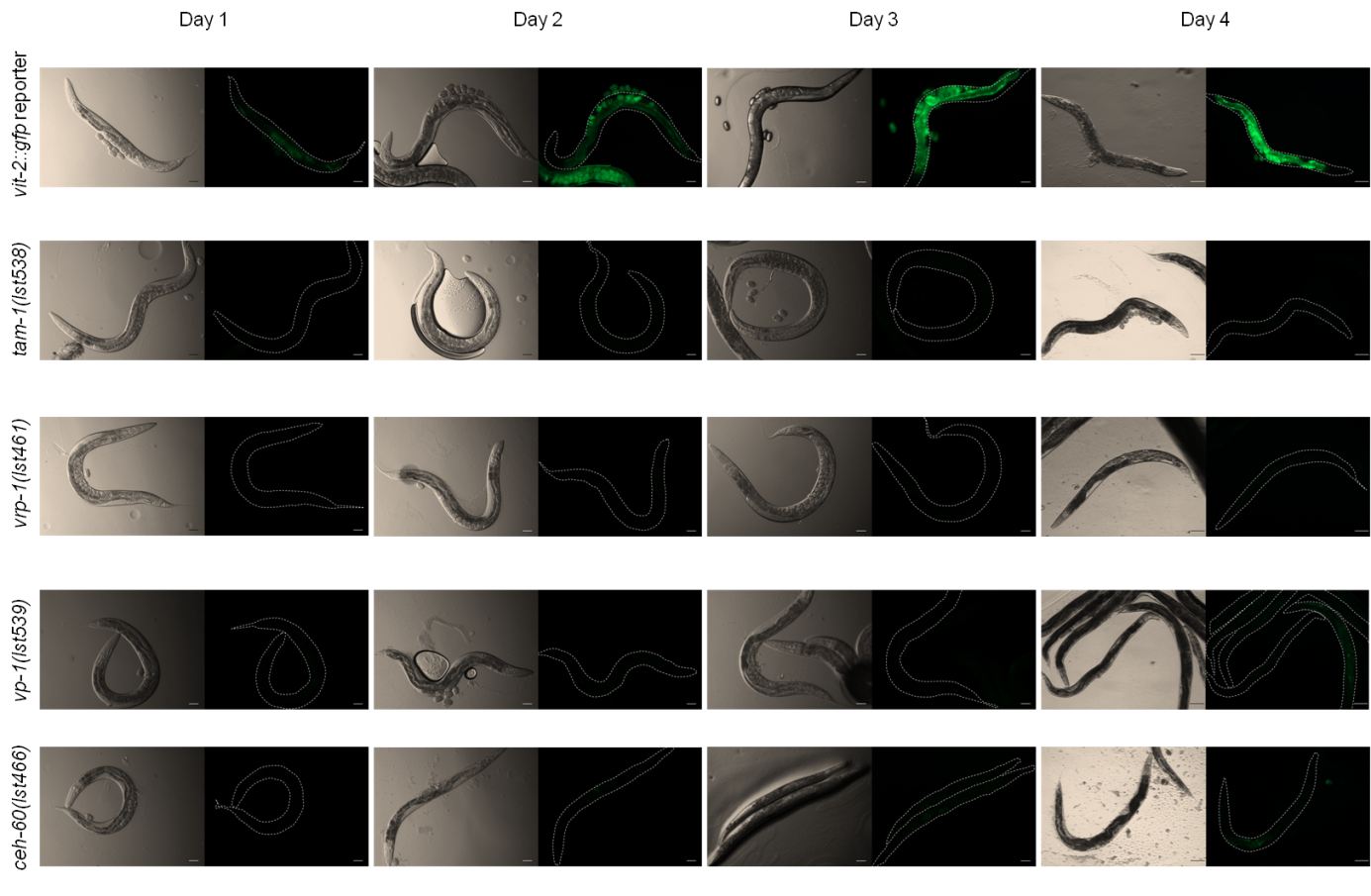
**

**
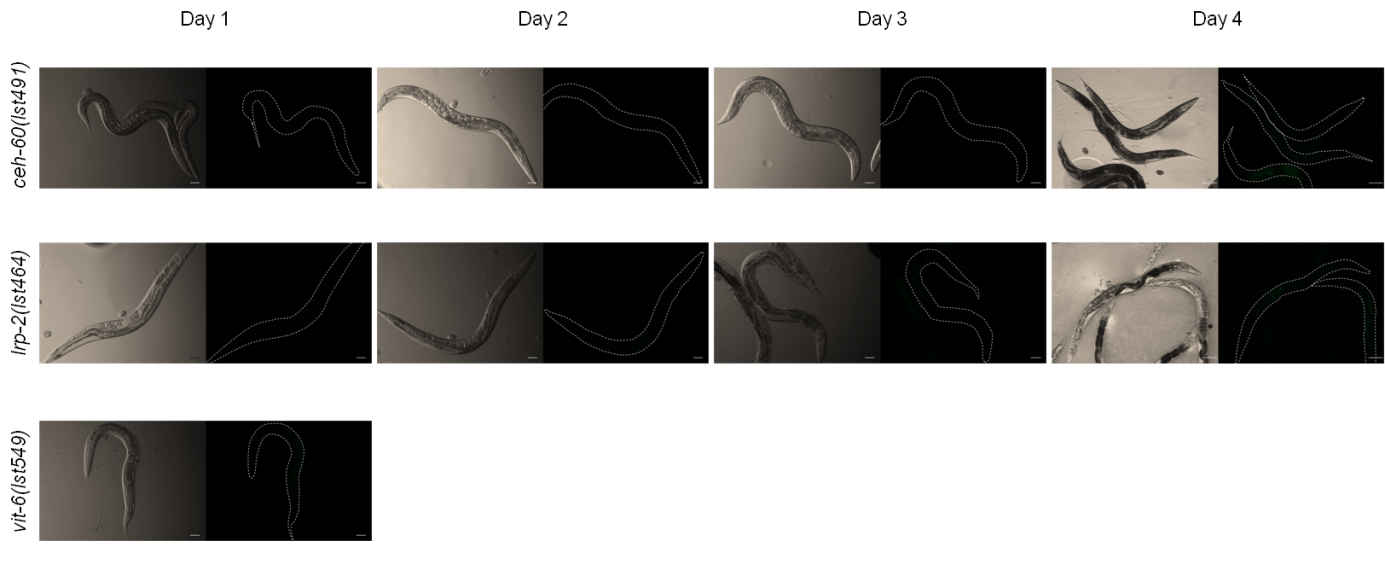
**

**Figure S2d**

**
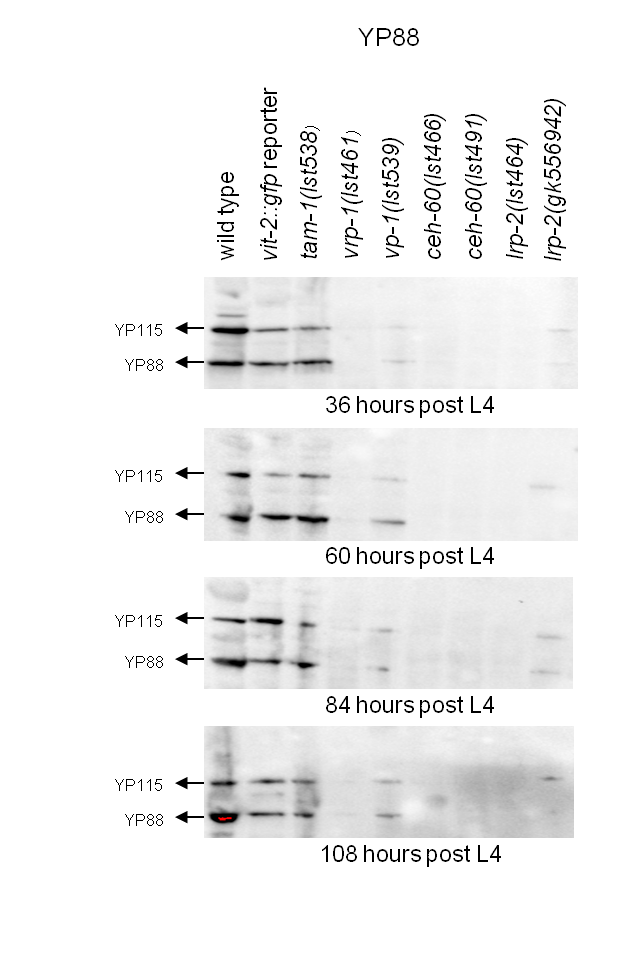
**

**Figure S2e**


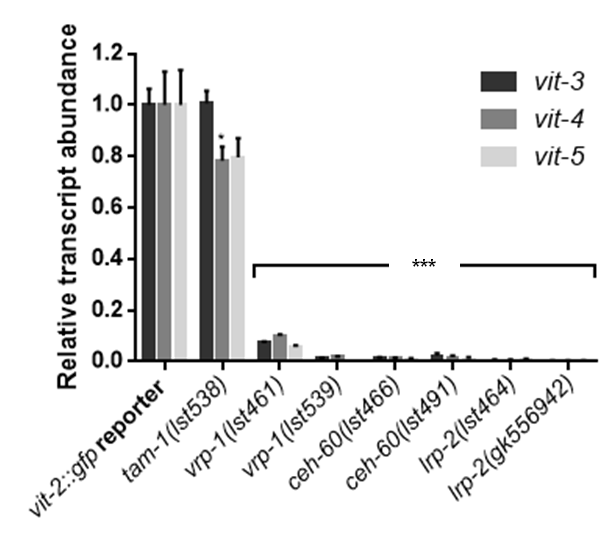


**Figure S2. Raw data underlying the relative quantification of yolk protein abundance in YPR mutants and controls, and additional quantification data.**

Representative images of SDS-PAGE analyses **(a)** clearly display compromised YP170 and YP170::GFP yolk protein pools in the YPR mutants compared to the *vit-2::gfp* reporter and wild-type controls, and relative to myosin abundance at the indicated time points (see also Fig. S3). **(b)** YP170::GFP yolk protein levels as analysed by SDS-PAGE were normalized against myosin levels (see also Fig. S3). For each indicated time point throughout reproductive development, the mean value of a maximum of four biologically independent measurements is plotted and connected to assist in overall profile evaluation. Compared to the *vit-2::gfp* reporter (
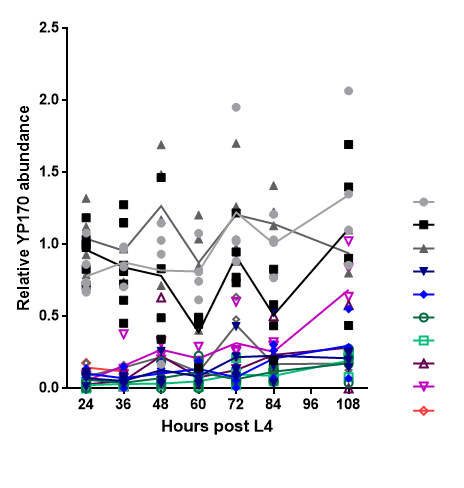
) and *tam-1(lst538)* (
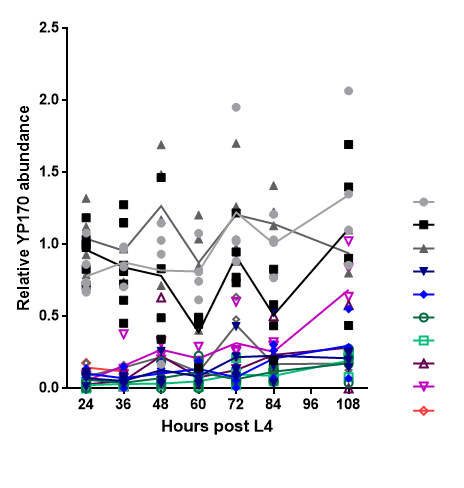
) controls, YP170::GFP yolk protein levels are practically absent in all YPR mutant populations, i.e. *vrp-1(lst461)* (
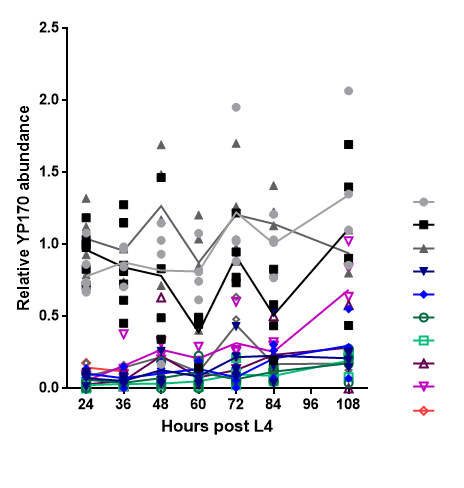
), *vrp-1(lst539)* (
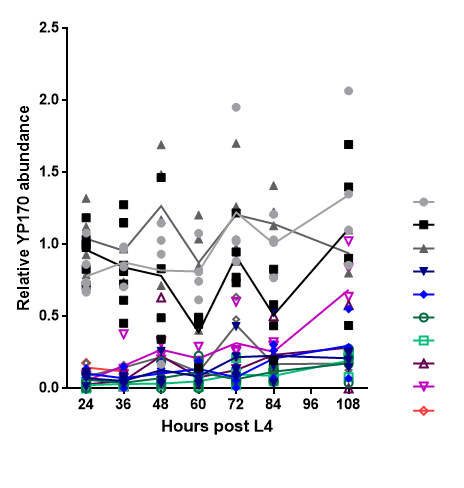
), *ceh-60(lst466)* (
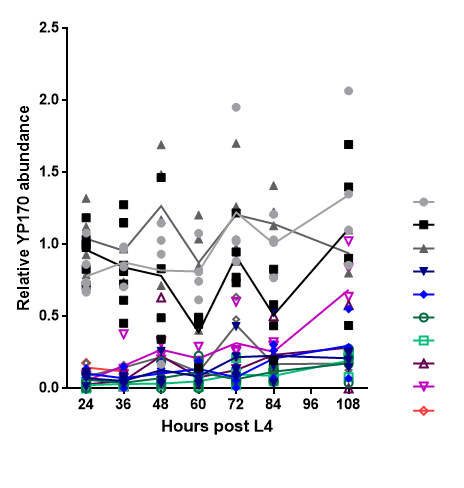
)*, ceh-60(lst491)* (
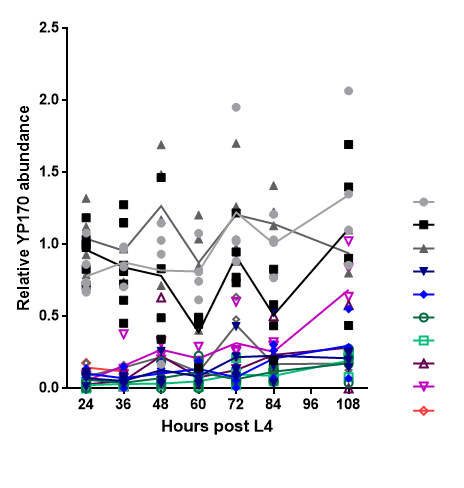
), *lrp-2(lst464)* (
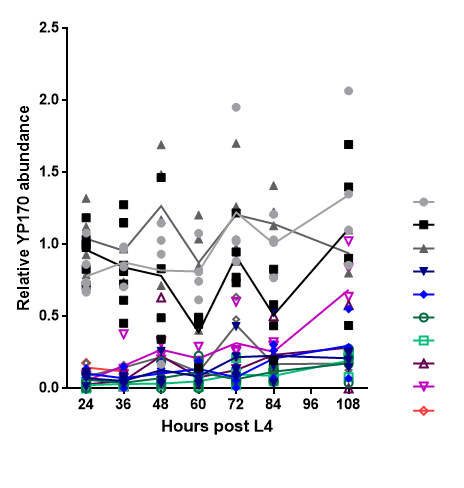
) and *lrp-2(gk556942)* (
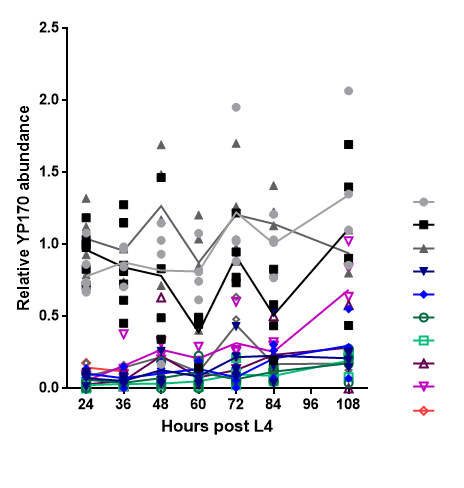
). **(c)** Representative images of control and YPR mutant *vit-2::gfp* signals as observed on days 1-4 of adulthood, which are in line with the protein data. Absence of a clear GFP signal is maintained throughout early adulthood for all YPR mutants, whereas bright VIT-2::GFP can persistently be detected in the reporter strain. Scale bar, 100 µm. **(d)** Representative immunoblot analyses reveal the YPR mutants’ affected YP88 yolk protein pools compared to the controls as normalized against each samples’ total protein stain. Furthermore, these immunoblot data seem to reveal cross-reactivity of the YP88 antibody against the YP115 yolk protein, which is similarly compromised in the YPR mutants. **(e)** Relative *vit-3, vit-4* and *vit-5* expression data for day 1 adult YPR mutants compared to the *vit-2::gfp* reporter and *tam-1(lst538)* mutant controls**.** Bars represent mean values ± SEM (n = 3) of *vit* transcript levels as measured by real-time PCR, relative to the *lstIs13 [vit-2::gfp]* positive control (****p* < 0.001 and **p* < 0.05). *vit-4* (**p* = 0.0303) transcript levels are slightly reduced in the semi-random *tam-1(lst538)* mutant control, though by far not to the same extent as in the YPR mutants. Please note that the method does not allow direct comparison of expression levels of different target genes within one sample.

**Figure S3a**


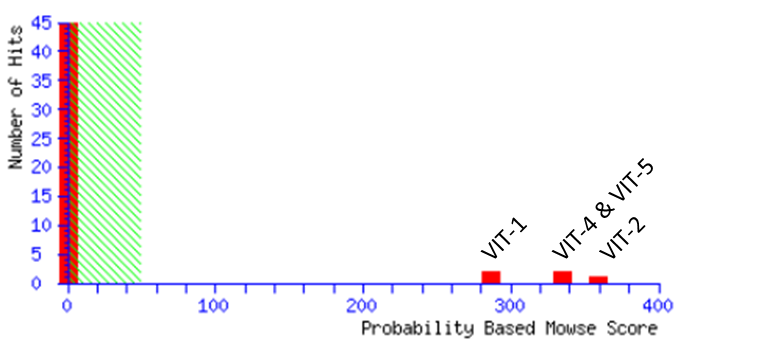


MRSIIIASLV ALALASSPAF ERTFEPKTDY HYK**FDGLVLS GLPSASSELS QSR**ISARAR**I QAVDDRYIHL QLVNIR**MAAS HLPESEQMPS LNSMEQR**ELS EEYKQMLELP LR**AQLR**NGLI SELQFDKEDA EWSK**NMKR**AV VNMISFNPIA PR**NEIEK**IES SYDKEEQSEE NTSFFTNEKT LEGDCQVAYT VIR**EQKKTII TKSINFDKCT ERSEIAYGLR YSSECPECEK DTELIRPQTV YTYVLENEEL KESEVRSLYT VNVNGQEVMK TETRSK**LVLE ENHSIK**SHIK KVNGEKESII YSSR**WEQLVE DFFK**NGDKAE FAPFEKFPLD KKMHLIK**TIT EQIQEVENNM PETSHFLAR**L VRIFRTTSTS QLKEIHETLY VKADKKIQSL MEHALAIAGT KNTIQHILVH MENEDILPLG QILK**TIQETP FPSQSIAEAL IK**FAESRVAK NNLVVR**QAAW LAAGSVVR**GI VDYKNIRPLV REDKRELKEK FLRVFMQQYK DAETTYEKIL ALK**TIGNAGL DISVNQLNEI IVDKR**QPLPV RKEAIDALRL LKDTMPRKIQ KVLLPIYKNR QYEPEIRMLA LWRMMHTRPE ESLLVQVVSQ MEK**ETNQQVA ALTHQMIR**HF AMSTNPCYQR VAIVCSKVLS FTRYQPQEQM IASSYAQLPL FLQNSFSGAQ FDFAAIFEKN SFLPKDLHAS LDAVFGGNWN KYFAQIGFSQ QHMDKYVQMA LEKLESLEKE STTVVRGRRI QTGIKLLKEL AQKMNIRARP ATYTEKDAFA MVYLRYKDMD YAFLPIDR**QL VENLIEK**FTS NGKVQFSEIR RLLNQELEFE THHAAYFYEA IR**KFPTTLGL PLTISGK**IPT VISAEGQFSL ELEGTELR**LT VEARPSVAAT HVYEMRMFTP LFEQGVK**SVQ SVRAYTPIKI QAVAGMKRNF EIVYKVVVPE NQKSIVSLTT RPVVFLRFPG FSK**FEYIEAE ERTVVVPQWQ QK**TQEIEK**VF NFLGLEVSTR** GNILNQHTLE NWLLAEQDFE VSVENKNRPA EFTARLTVGQ LEKTELSQIK YNKIFEKEFE LEQENTESRR EYFNKMVKNI QKEQGYKSVI SLKLEAPRDY TMNTELTTVC DKQVRMCQWE VEIRR**SPILE ETKEWTLRSQ LLVVRPEMPS SLR**QLRDQPH R**EVQLSLTST WGSQK**K**SEVT VNAQLQQSK**E QKKYERNMDR **QFNGMPEYEL LIK**AAR**LNQI NAVAEYK**LTR ETEQVLARYF DLVKTYNYWT VSSRPENNEN DR**VVVQLTVE PMSR**QYVNIT MQSPMERIEL KNVQVPR**VYL PSIAQR**SVKH QLTEASGSVC KVQKNQIR**TF DDVLYNTPLT TCYSLIAK**DC SEEPTFAVLS KKTEKNSEEM IIKVIR**GEQE IVAQLQNEEI R**VKVDGKK**IQ SEDYSAYQIE RLGESAIVIE LPEGEVR**FDG YTIKTQLPSY SRKNQLCGLC GNNDDESTNE FYTSDNTETE DIEEFHRSYL LKNEECEAEE ERLSEKKNYR KYERDEEQSD EYSSEETYDY EQENTKKSQK NQRSQKKSDL VEKTQIKEFS HR**ICFSVEPV AECR**RGYEVE QQQQRKIRFT CLQRHNRDAS RLLKESRQQP LQLDDYPVSF VESVKVPTAC VAY

**Figure S3b**

**
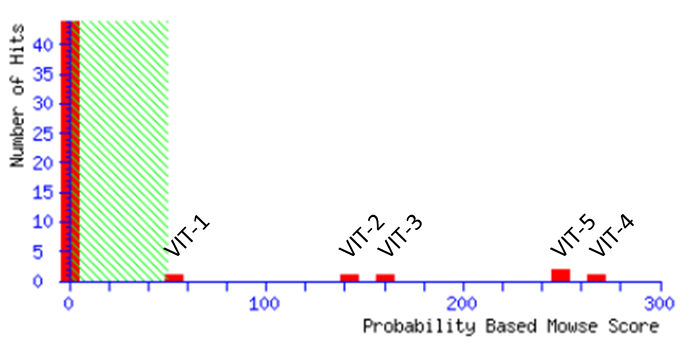
**

MKSIIIASLV ALAIAASPAL DRTFSPKSEY VYKFDGLLLS GLPTTFSDAS QTLISCRTRL QAVDDRYIHL QLIDIQYSAS HIPQSEQWPK IESLEQRELS DELK**ELLELP FR**AQIRNGLV SEIQFSSEDA EWSKNAKR**SI LNLFSLR**KSA PVDEMSQDQK DMESDKDSLF FNVHEKTMEG DCEVAYTIVQ EGGKTIYTKS VNFDK**CITRP ETAYGLR**FGS ECKECEK**EGQ FVQPQTVYTY TFK**NEKLQES EVNSIYTLNV NGQEVVKSET RAKVTFVEES KINREIKKVS GPKEEIVYSM ENEK**LIEQFY KQGDK**AEVNP FKAIEIEQK**V EQLEEIFRQI QEHEQNTPET VHLIAR**AVRM FRMCTIEELK KVHTTIYTKA EKKVQLVIET TLAVAGTKNT IQHLIHHFEK KSITPLRAAE LLKSVQETLY PSEHIADLLI QLAQSPLSEK YEPLR**QSAWL AAGSVVR**GFA SKTQDLPLIR PASRQTKEKY VRVFMQHFRN ADSTYEKVLA LKTLGNAGID LSVYELVQLI QDPRQPLSIR TEAVDALRLL KDVMPRKIQK VLLPVYKNRQ NKPELRMAAL WRMMHTIPEE PVLAHIVSQM ENESNQHVAA FTYNVLRQFS K**STNPCYQQL AVR**CSKVLLF TRYQPQEQML STYSQLPLFN SEWLSGVQFD FATIFEKNAF LPKEVQASFE TVFGGNWNKY FAQVGFSQQN FEQVILKTLE KLSLYGKQSD ELRSRR**VQSG IQMLQEIVK**K MNIRPR**VQQT DSQNAHAVFY LR**YKEMDYIV LPIDMETIDN VVEKYVRNGE FDIK**SLLTFL TNDSK**FELHR **ALFFYEAER**R **IPTTIGMPLT ISGK**MPTILS INGKVSIELE KLGARLVLDI VPTVATTHVT EMR**FWYPVIE QGVK**SLQSAR LHTPLRFEST VELKKNTLEI THKFVVPENK KTTVSVHTRP VAFIRVPKNQ DSEYVEAEEK **TISHSQYQMS TEEIDR**QYET FGLRINAQGN VLSQWTLPMV LMTEQDFEFT LENKNRPVEF TARVTIGNLE K**TDLSEIKFD K**IFEKEFDLE NNESENRRQY FHKMIREIQS EQGFKNLITL K**LEAPQQMYW NTELR**TVCDK WIRMCKVEMD ARRSPIEHEN KEWTLR**TELL AARPQMPSSL R**QLREQPHRE VQLALNAKWG SSKKSEITFN AQLEQSTEQK KFLRNIEREY K**GIPEYELLI K**AAR**LNQVNV VSEYKLTPES EYTFSR**IFDL IK**AYNFWTVS EK**RVQNEDRR **VVLQLSVEPL SR**QYMNMTIQ TPEQEVELKN VRIPR**VVLPT IAR**RAMFQQT WEKTGATCK**V DQSEVSTFDN VIYRAPLTTC YSLVAK**DCSE QPRFAVLAKK INKNSEELLV KVVRREEEIV VKKSDDKFLV KVDGKKVNPT ELEQYNIEIL GDNLIVIRLP HGEVRFDGYT VKTNMPSVAS QNQLCGLCGN NDGERDNEFM TADNYETEDV EEFHRSYLLK NEECEVEKDR ISEKKNYKNK WNREEKKSDY ESSSDYESNY DEKETEKELV KKTLIKEFSN R**VCFSIEPVS ECR**RGLESEK TSNKKIRFTC MPRHSKNARR FLKEAREQTV ADLVDFPVSF VESVKIPTAC VAY

**Figure S3c**

**
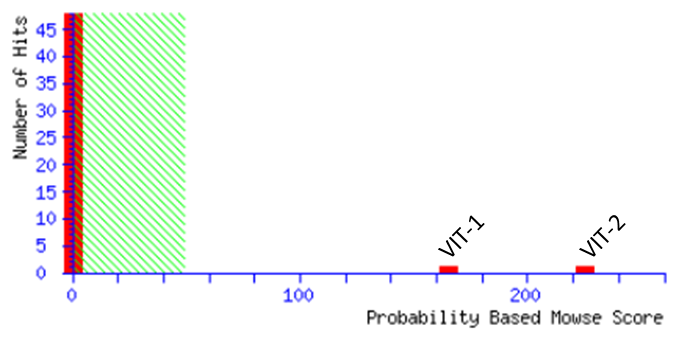
**

MRSIIIASLV ALALASSPAF ERTFEPKTDY HYK**FDGLVLS GLPSASSELS QSR**ISARAR**I QAVDDRYIHL QLVNIR**MAAS HLPESEQMPS LNSMEQR**ELS EEYKQMLELP LR**AQLRNGLI SELQFDKEDA EWSKNMKR**AV VNMISFNPIA PR**NEIEK**IES SYDKEEQSEE NTSFFTNEKT LEGDCQVAYT VIR**EQKKTII TKSINFDKCT ERSEIAYGLR YSSECPECEK DTELIRPQTV YTYVLENEEL KESEVRSLYT VNVNGQEVMK TETRSKLVLE ENHSIKSHIK KVNGEKESII YSSR**WEQLVE DFFK**NGDKAE FAPFEKFPLD KKMHLIK**TIT EQIQEVENNM PETSHFLAR**L VRIFRTTSTS QLKEIHETLY VKADK**KIQSL MEHALAIAGT K**NTIQHILVH MENEDILPLG QILKTIQETP FPSQSIAEAL IKFAESRVAK NNLVVR**QAAW LAAGSVVR**GI VDYKNIRPLV REDKRELKEK FLRVFMQQYK DAETTYEKIL ALKTIGNAGL DISVNQLNEI IVDKRQPLPV RKEAIDALRL LKDTMPRKIQ KVLLPIYKNR QYEPEIRMLA LWRMMHTRPE ESLLVQVVSQ MEK**ETNQQVA ALTHQMIR**HF AMSTNPCYQR VAIVCSKVLS FTRYQPQEQM IASSYAQLPL FLQNSFSGAQ FDFAAIFEKN SFLPKDLHAS LDAVFGGNWN KYFAQIGFSQ QHMDKYVQMA LEKLESLEKE STTVVRGRRI QTGIKLLKEL AQKMNIRARP ATYTEKDAFA MVYLRYKDMD YAFLPIDR**QL VENLIEK**FTS NGKVQFSEIR RLLNQELEFE THHAAYFYEA IR**KFPTTLGL PLTISGK**IPT VISAEGQFSL ELEGTELRLT VEARPSVAAT HVYEMR**MFTP LFEQGVK**SVQ SVRAYTPIKI QAVAGMKRNF EIVYKVVVPE NQK**SIVSLTT RPVVFLR**FPG FSK**FEYIEAE ER**TVVVPQWQ QKTQEIEK**VF NFLGLEVSTR** GNILNQHTLE NWLLAEQDFE VSVENKNRPA EFTARLTVGQ LEKTELSQIK YNKIFEKEFE LEQENTESRR EYFNKMVKNI QKEQGYKSVI SLKLEAPRDY TMNTELTTVC DKQVR**MCQWE VEIR**R**SPILE ETKEWTLRSQ LLVVRPEMPS SLR**QLRDQPH R**EVQLSLTST WGSQK**K**SEVT VNAQLQQSK**E QKKYERNMDR QFNGMPEYEL LIKAAR**LNQI NAVAEYK**LTR ETEQVLARYF DLVKTYNYWT VSSRPENNEN DR**VVVQLTVE PMSR**QYVNIT MQSPMERIEL KNVQVPR**VYL PSIAQR**SVKH QLTEASGSVC KVQKNQIRTF DDVLYNTPLT TCYSLIAKDC SEEPTFAVLS KKTEKNSEEM IIKVIR**GEQE IVAQLQNEEI R**VKVDGKK**IQ SEDYSAYQIE RLGESAIVIE LPEGEVR**FDG YTIKTQLPSY SRKNQLCGLC GNNDDESTNE FYTSDNTETE DIEEFHRSYL LKNEECEAEE ERLSEKKNYR KYERDEEQSD EYSSEETYDY EQENTKKSQK NQRSQKKSDL VEKTQIKEFS HR**ICFSVEPV AECR**RGYEVE QQQQRKIRFT CLQRHNRDAS RLLKESR**QQP LQLDDYPVSF VESVK**VPTAC VAY

**Figure S3d**

**
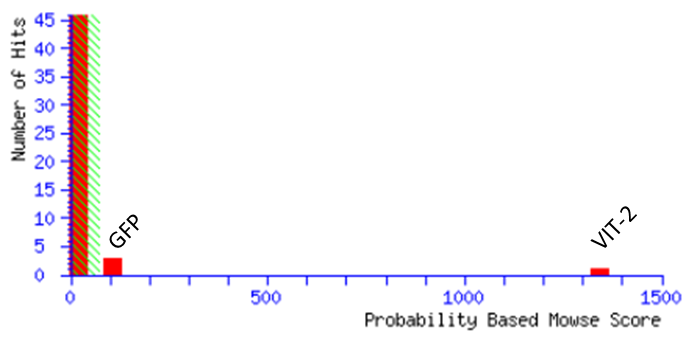
**

MSKGEELFTG VVPILVELDG DVNGHK**FSVS GEGEGDATYG K**LTLKFICTT GKLPVPWPTL VTTFSYGVQC FSRYPDHMKQ HDFFKSAMPE GYVQERTIFF KDDGNYKTRA EVKFEGDTLV NRIELK**GIDF KEDGNILGHK LEYNYNSHNV YIMADK**QKNG IKVNFKIRHN IEDGSVQLAD HYQQNTPIGD GPVLLPDNHY LSTQSALSKD PNEKRDHMVL LEFVTAAGIT HGMDELYK

**Figure S3. Mascot MS/MS analysis.**

Results of MASCOT MS/MS ion searches performed upon ESI-QTOF runs of excised bands of suspected YP170 in Fig. S2. Note that *vit-1* through *vit-5* genes encode YP170 proteins, whereas *vit-6* encodes YP115 and YP88 (hence, is not detected in the bands analysed here). **(a)** Wild type endogenous YP170 band: Ions score is -10*Log(P), where P is the probability that the observed match is a random event. Individual ions scores > 49 indicate identity or extensive homology (*p* < 0.0001). Protein scores are derived from ions scores as a non-probabilistic basis for ranking protein hits. VIT-2 sequence coverage by tryptic digested peptides in this sample: 30% (39 queries matched); VIT-4 sequence coverage by tryptic digested peptides in this sample: 28% (43 queries matched); VIT-5 sequence coverage by tryptic digested peptides in this sample: 31% (43 queries matched); VIT-1 sequence coverage by tryptic digested peptides in this sample: 23% (29 queries matched). Top hit (VIT-2) matched peptides are shown in bold red. **(b)** *vit-2::gfp* reporter strain endogenous YP170 band: Ions score is -10*Log(P), where P is the probability that the observed match is a random event. Individual ions scores > 50 indicate identity or extensive homology (*p* < 0.0001). Protein scores are derived from ions scores as a non-probabilistic basis for ranking protein hits. VIT-4 sequence coverage by tryptic digested peptides in this sample: 21% (31 queries matched); VIT-5 sequence coverage by tryptic digested peptides in this sample: 23% (32 queries matched); VIT-3 sequence coverage by tryptic digested peptides in this sample: 21% (31 queries matched); VIT-2 sequence coverage by tryptic digested peptides in this sample: 21% (28 queries matched); VIT-1 sequence coverage by tryptic digested peptides in this sample: 16% (21 queries matched). Top hit (VIT-4) matched peptides are shown in bold red. **(c)** *vit-2::gfp* reporter strain reporter YP170-GFP band: Ions score is -10*Log(P), where P is the probability that the observed match is a random event. Individual ions scores > 49 indicate identity or extensive homology (*p* < 0.0001). Protein scores are derived from ions scores as a non-probabilistic basis for ranking protein hits. VIT-2 sequence coverage by tryptic digested peptides in this sample: 25% (39 queries matched); VIT-1 sequence coverage by tryptic digested peptides in this sample: 16% (27 queries matched). Top hit (VIT-2) matched peptides are shown in bold red. **(d)** Please note that MS/MS data were analysed against SwissProt’s *C. elegans* entries, therefore, GFP is not identified. Rerunning these data against all SwissProt entries, results in considerable detection of both VIT-2 and GFP. VIT-2 sequence coverage by tryptic digested peptides in this sample: 25% (39 queries matched); GFP sequence coverage by tryptic digested peptides in this sample: 18% (3 queries matched). GFP matched peptides shown in bold red.

**Figure S4**

**
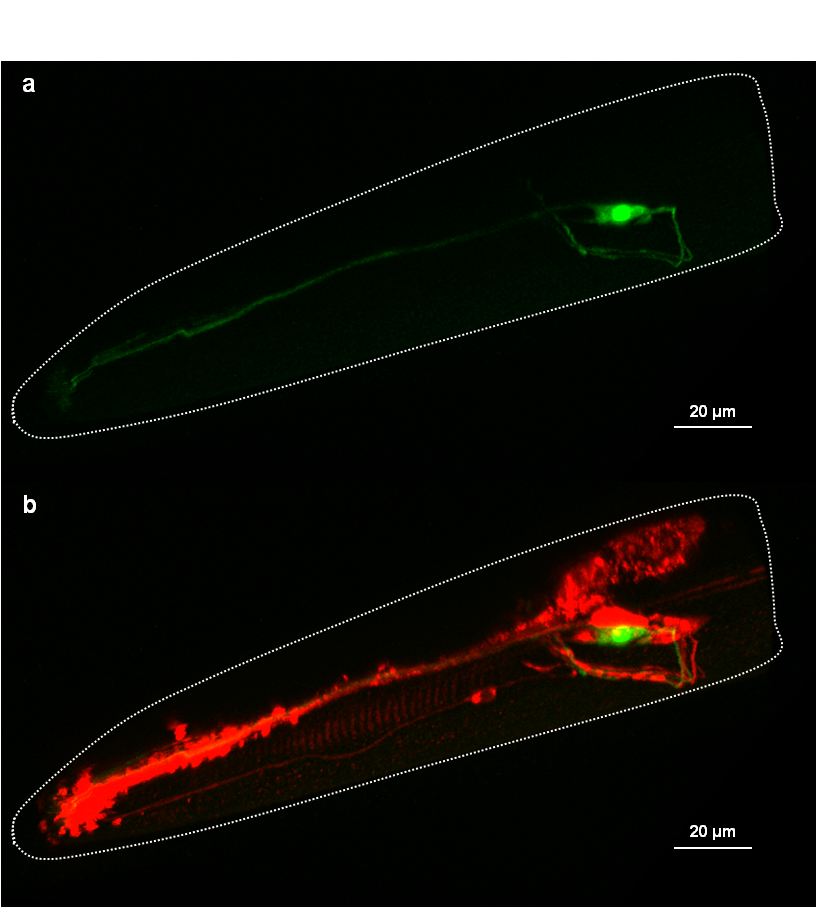
**

**Figure S4. Confirmation of amphidial *ceh-60* expression.**

Using **(a+b)** *Pceh-60::gfp* and **(b)** DiI staining as a localization marker, we confirmed the earlier reported expression of this construct ^2^ in a single pair of amphid neurons. In addition, but not visible in this figure, we occasionally observed weak expression in a second pair of amphids.

**Figure S5**

**
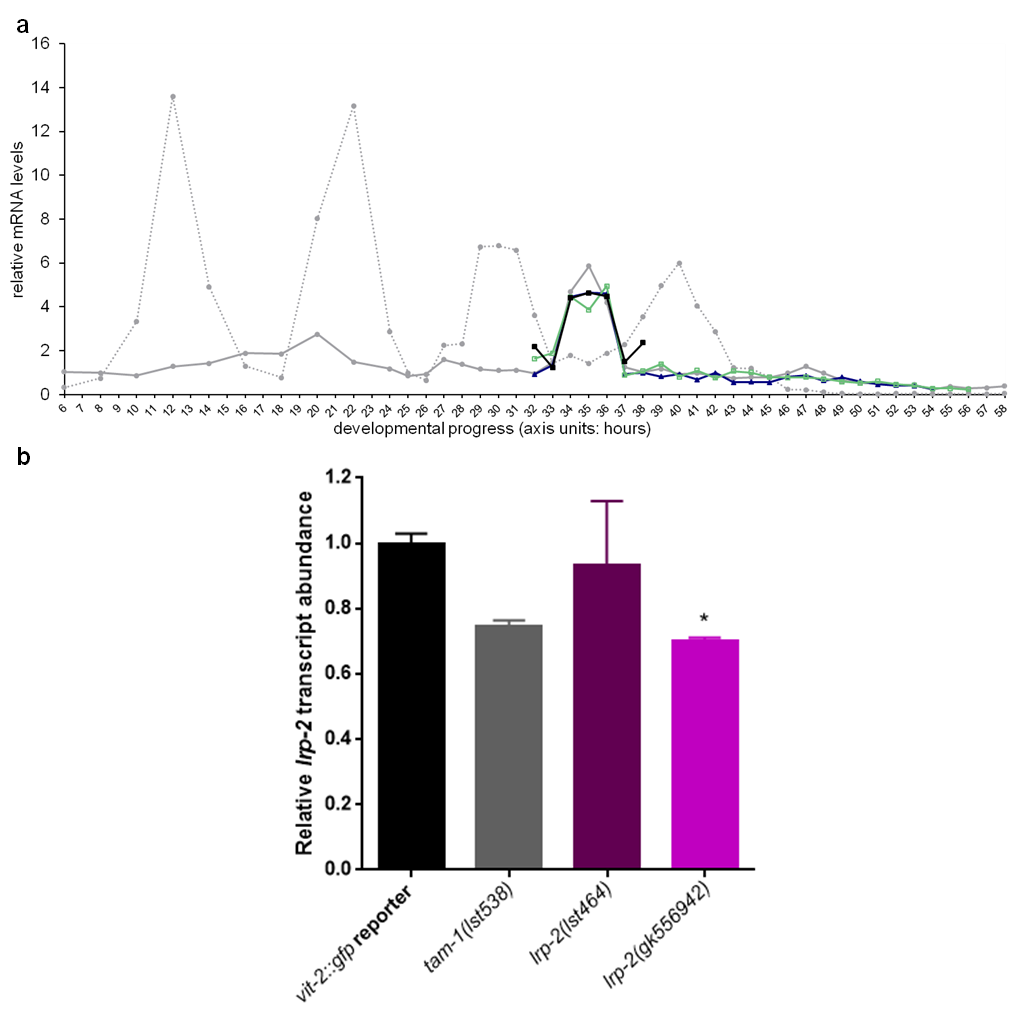
**

**Figure S5. *lrp-2* gene profile during development.**

The light grey dotted line represents the wild-type *lin-42* profile, which was generated to assist in developmental timing evaluation ^3^. **(a)** *lrp-2* expression in the following strains: wild type (
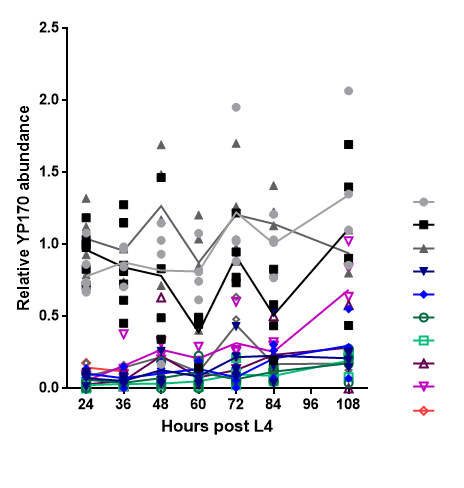
), reporter control (
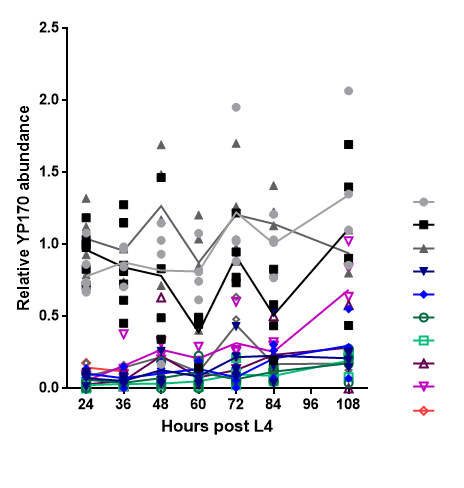
), *vrp-1(lst461)* (
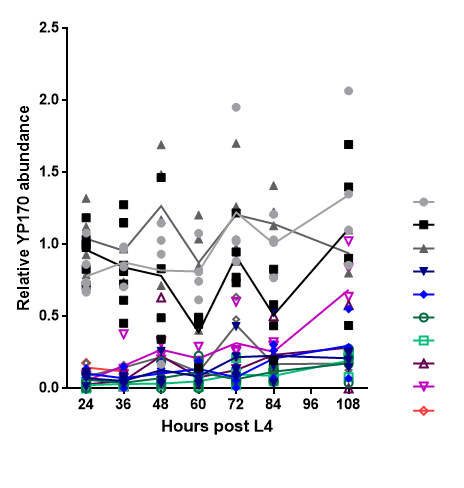
), *ceh-60(lst491)* (). **(b)** Relative expression data for day 1 adult hermaphrodites. (a+b) *lrp-2* expression remains unaltered in all mutants, including its own (see Discussion for details on *lrp-2* expression data).


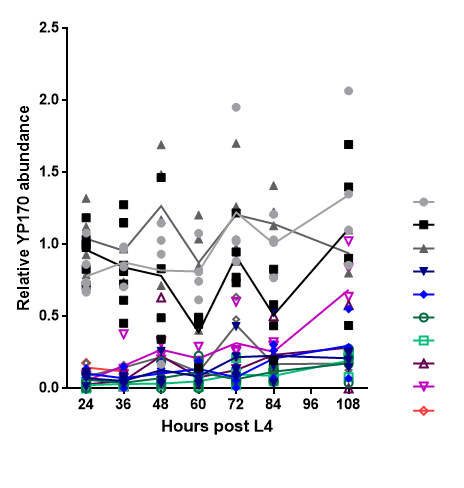


**Figure S6**

**
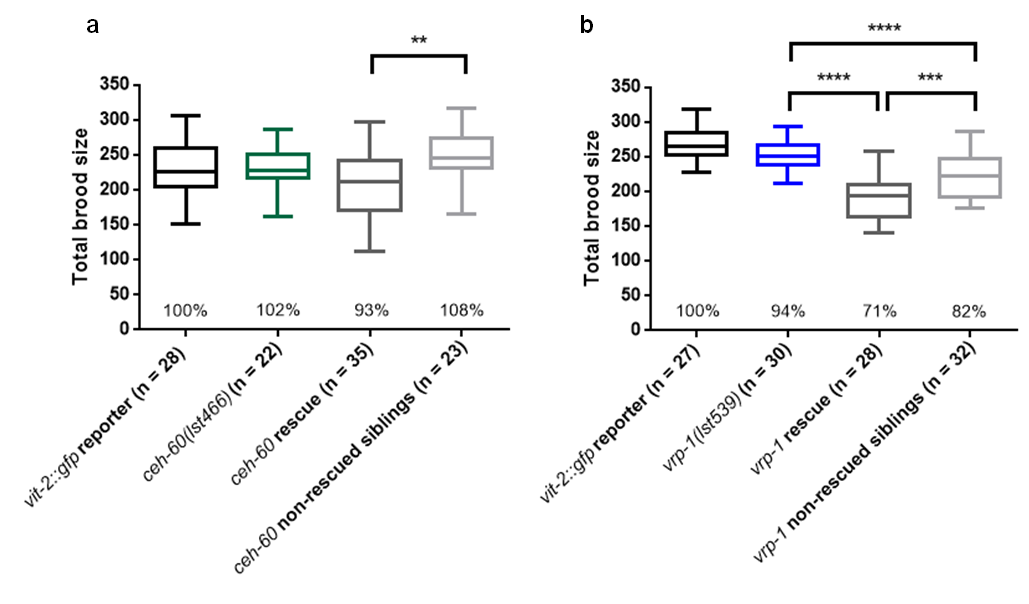
**

**Figure S6.** **Total brood size of YPR mutants.**

Box plots reflect the mean total brood sizes ± SEM (n = number of adults evaluated) of the YPR mutants **(a)** *vrp-1(lst539)* and **(b)** *ceh-60(lst466)*, their rescues and corresponding (non-rescued) siblings, compared to the *vit-2::gfp* reporter control (***p* < 0.01, ****p* < 0.001, *****p* < 0.0001).

**Figure S7**


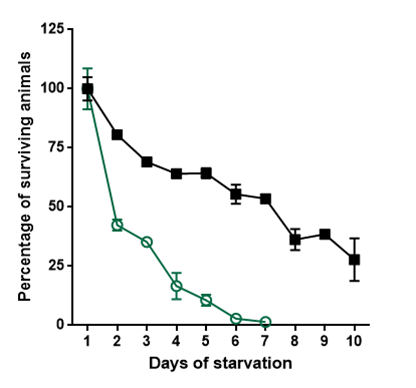


**Figure S7. L1 diapause survival is consistently affected in *ceh-60(lst466)* mutants.** Supporting the observations using a density of 11 worms/µl, also at 5 worms/µl *ceh-60(lst466)* (
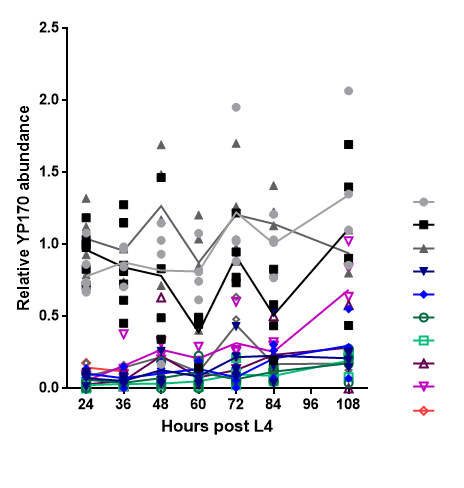
) individuals cannot cope with survival in absence of food, as opposed to the *vit-2::gfp* reporter control (
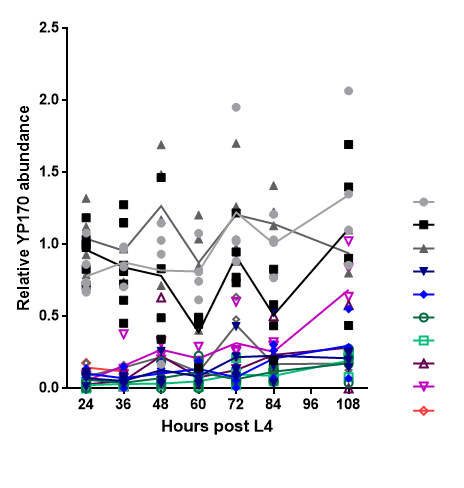
). Because of the density dependency of the assay, differences in worm density absolute values presented here cannot be directly compared with those in Fig. 6 ^4^.

**Supplementary Tables S1-7**

**Genetic regulators and relevance of abundant yolk protein production in *C. elegans***

**Liesbeth Van Rompay, Charline Borghgraef, Isabel Beets, Jelle Caers and Liesbet Temmerman***

Functional Genomics and Proteomics

Department of Biology, KU Leuven

3000 Leuven, Belgium

*Corresponding author: Liesbet.Temmerman@bio.kuleuven.be

**Table S1. List of all annotated homozygous variants for each LSC mutant under consideration, according to** ^5^**.** *See uploaded file*

**Table S2. Biologically independent measurements of endogenous YP170 and YP88 yolk protein, and *vit-2*, *vit-3* and *vit-6* mRNA levels in selected YPR mutants and controls.** *See uploaded file*

**Table S3. Gene expression patterns and stages of *vrp-1*, *ceh-60* and *lrp-2***.

| **Gene** | **Expression site** | **Life stage** | **Reference** |
| --- | --- | --- | --- |
| *vrp-1* | Intestine | L1-adult | This study |
| *ceh-60* | Amphid neurons | L1-adult | 2 |
| *lrp-2* | Pharynx, intestine, uterine muscle, vulval muscle, body wall muscle, hypodermis, excretory cell, head neuron, ventral cord neuron, tail neuron and tail | Larva-adult | 6 |

Table S4. Oligonucleotides (5’-3’) used for genotype confirmation.

| **Gene(allelic variant)** | **PCR forward primer sequence** | **PCR reverse primer sequence** | **Sanger sequencing primer sequence** |
| --- | --- | --- | --- |
| *tam-1(lst538)* | ACGTGGCCCACTTCTACATC | ATGTGCTGAGCATGAACTCG | GAGACGTGGACCAGAAGCTC |
| *vrp-1(lst461, lst539)* | GCTGATTGGTTTCGCATTTT | TCTTTCATGGACGGCTTCTT | GAGCGAAACAACGAATGGAT |
| *ceh-60(lst466)* | GAGTACGTGGGCAGCAAAAA | GATTTTGCCAATTCGCATCT | CTGACCGTAGATCGAACGAA |
| *ceh-60(lst491)* | CCGATCGACGAAAAGGTAAA | TTGCGTCTGAAACAAAATCG | TGAAACGATCGAGATCATCA |
| *lrp-2(lst464)* | AGGAATGCGCAATTGTTTGT | CCTGCCGATCAAACAAGACT | GCTCGATGCAAACAATCAAA |
| *lrp-2(gk556942)* | GCACGTCGTCTGGAAAATCT | CCGAGTGCAATGACTTTTGA | CATGACTGATCAACGACGAA |
| *gfp* | GCCCGAAGGTTATGTACAGG | AAAGGGCAGATTGTGTGGAC | / |

Table S5. Oligonucleotides (5’-3’) used for genomic and cDNA rescue constructs.

| **Genomic rescue** | **PCR forward primer sequence** | **PCR reverse primer sequence** |
| --- | --- | --- |
| *vrp-1* | GGCCTATTTCGGCAAACTCT | CTCCGCAGCTTCCATTATTC |
| *ceh-60* | GTAGGCGAAGAGGTCAGTGG | AACTTGTCCTGTCGGTCCAG |
| *lrp-2* | GTCGCCCTCATCCTTCTTTT | TCGTCGGGTCTATGAAGCTC |
| *oac-2* | GGAAAACATGGAAAGCCAAA | GAAGTTGCCAAAAACGGATT |
| *T14A8.2* | AGACGCCAATTTTTGTCTGA | CCCCACTGGCTCCACTATTA |
| **cDNA rescue** | **PCR forward primer sequence^a^** | **PCR reverse primer sequence^a^** |
| *vrp-1* promoter | GGCCTATTTCGGCAAACTCT | AGCTGTTCCTCGTTGATCATTATGCAATTATGGGGTGCGA |
| *vrp-1* cDNA | TCGCACCCCATAATTGCATAATGATCAACGAGGAACAGCT | **AAAAAAAAAGAAGTTATTTTTCATTCCAAGTCATCCAAAT** |
| *vrp-1* 3’ UTR | **ATTTGGATGACTTGGAATGAAAAATAACTTCTTTTTTTTT** | CTCCGCAGCTTCCATTATTC |

^a^Overlapping regions in forward and reverse primer sequences are underlined or indicated in bold.

Table S6. Oligonucleotides (5’-3’) used to create the VRP-1 localization construct.

| **Gene** | **PCR forward primer sequence^a^** | **PCR reverse primer sequence^a^** |
| --- | --- | --- |
| *vrp-1* | GCCGTAAATCTACCCCAGAT | **AGTCGACCTGCAGGCATGCAAGCT**TTCCAAGTCATCCAAATCTCTC |
| *gfp* | **AGCTTGCATGCCTGCAGGTCGACT** | AAGGGCCCGTACGGCCGACTAGTAGG |
| *vrp-1::gfp* | GGCCTATTTCGGCAAACTCT (nested) | GGAAACAGTTATGTTTGGTATATTGGG (nested) |

^a^Overlapping regions in forward and reverse primer sequences are indicated in bold.

Table S7: Oligonucleotides (5’-3’) used for real-time PCR.

| **Gene** | **PCR forward primer sequence** | **PCR reverse primer sequence** | **Reference** |
| --- | --- | --- | --- |
| *vit-2* | GACACCGAGCTCATCCGCCCA | TTCCTTCTCTCCATTGACCT | 7 |
| *vit-3* | TCTGAGACTCGCTCTAAGGTCAC | GAGTAGACGATCTCCTCCTTTGG | 8 |
| *vit-4* | AGAGGTTGTCAAGTCTGAGACTC | CGATAGCCTTGAATGGGTTGAC | 8 |
| *vit-5* | GGCAATTTGTTAAGCCACAA | CCTCCTTTGGTCCAGAAACCT | 7 |
| *vit-6* | CCAAGAGAACACCATTCCAAA | CTCCTCTTGATTTTTCTCAATGC | 8 |
| *vrp-1* | CAGAAGATGCCGAGTGGTGA | TTGAGAGCTTCTAGTTTGTACGGATC | This study |
| *ceh-60* | AGGAAATTCTCGCTCAATTCGA | TGACCTCTTCCAACGGAAATG | This study |
| *lrp-2* | TCAGGGTTTCAGCCTCGTTC | TGGGTTTCCTCCGCTGAC | This study |
| *cdc-42* | AGCCATTCTGGCCGCTCTCG | GCAACCGCTTCTCGTTTGGC | 9 |
| *pmp-3* | TGGCCGGATGATGGTGTCGC | ACGAACAATGCCAAAGGCCAGC | 9 |
| *tba-1* | TCAACACTGCCATCGCCGCC | TCCAAGCGAGACCAGGCTTCAG | 9 |

**Supplementary References**

1. Thompson, O. *et al.* The million mutation project: a new approach to genetics in *Caenorhabditis elegans*. *Genome Res.* **23,** 1749–1762 (2013).

2. Reece-Hoyes, J. S. *et al.* Insight into transcription factor gene duplication from *Caenorhabditis elegans* Promoterome-driven expression patterns. *BMC Genomics* **8,** 27 (2007).

3. Jeon, M., Gardner, H. F., Miller, E. A., Deshler, J. & Rougvie, A. E. Similarity of the *C. elegans* developmental timing protein LIN-42 to circadian rhythm proteins. *Science* **286,** 1141–1146 (1999).

4. Artyukhin, A. B., Schroeder, F. C. & Avery, L. Density dependence in *Caenorhabditis* larval starvation. *Sci. Rep.* **3,** 2777 (2013).

5. Minevich, G., Park, D. S., Blankenberg, D., Poole, R. J. & Hobert, O. CloudMap: a cloud-based pipeline for analysis of mutant genome sequences. *Genetics* **192,** 1249–1269 (2012).

6. McKay, S. J. *et al.* Gene expression profiling of cells, tissues, and developmental stages of the nematode *C. elegans*. *Cold Spring Harb. Symp. Quant. Biol.* **68,** 159–169 (2003).

7. DePina, A. S. *et al.* Regulation of *Caenorhabditis elegans* vitellogenesis by DAF-2/IIS through separable transcriptional and posttranscriptional mechanisms. *BMC Physiol.* **11,** 11 (2011).

8. Jovanovic, M. *et al.* A quantitative targeted proteomics approach to validate predicted microRNA targets in *C. elegans*. *Nat. Methods* **7,** 837–842 (2010).

9. Hoogewijs, D., Houthoofd, K., Matthijssens, F., Vandesompele, J. & Vanfleteren, J. R. Selection and validation of a set of reliable reference genes for quantitative *sod* gene expression analysis in *C. elegans*. *BMC Mol. Biol.* **9,** 9 (2008).
